# Ambient Processed rGO/Ti_3_CNT_*x*_ MXene Thin Film with High Oxidation Stability, Photosensitivity, and Self-Cleaning Potential

**DOI:** 10.1021/acsami.3c07972

**Published:** 2023-09-08

**Authors:** Muhammad Abiyyu Kenichi Purbayanto, Dominika Bury, Madhurya Chandel, Zhila Dehghan Shahrak, Vadym N. Mochalin, Anna Wójcik, Dorota Moszczyńska, Anita Wojciechowska, Anika Tabassum, Michael Naguib, Agnieszka Maria Jastrzębska

**Affiliations:** † Faculty of Materials Science and Engineering, Warsaw University of Technology, Wołoska 141, Warsaw 02-507, Poland; ‡ Department of Chemistry, Missouri University of Science and Technology, Rolla, Missouri 65409 United States; § Department of Materials Science and Engineering, Missouri University of Science and Technology, Rolla, Missouri 65409 United States; ∥ Polish Academy of Sciences, Institute of Metallurgy and Materials Science, W. Reymonta 25, 30-059 Cracow, Poland; ⊥ Department of Physics and Engineering Physics, Tulane University, New Orleans, Louisiana 70118, United States

**Keywords:** Ti_3_CNT_*x*_ MXene, stability, conductivity, self-cleaning, photocatalytic

## Abstract

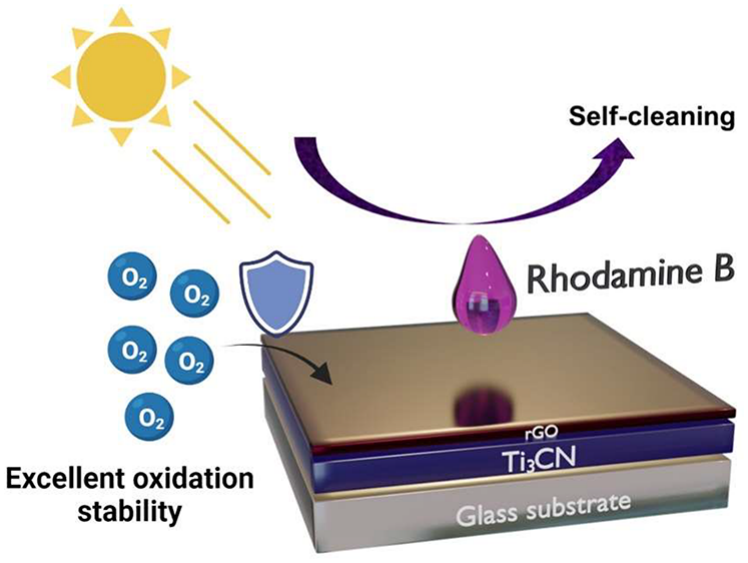

Solution-based processing offers advantages for producing thin films due to scalability, low cost, simplicity, and benignity to the environment. Here, we develop conductive and photoactivated self-cleaning reduced graphene oxide (rGO)/Ti_3_CNT_*x*_ MXene thin films *via* spin coating under ambient conditions. The addition of a thin rGO layer on top of Ti_3_CNT_*x*_ resulted in up to 45-fold improvement in the environmental stability of the film compared to the bare Ti_3_CNT_*x*_ film. The optimized rGO/Ti_3_CNT_*x*_ thin film exhibits an optical transmittance of 74% in the visible region of the spectrum and a sheet resistance of 19 kΩ/sq. The rGO/Ti_3_CNT_*x*_ films show high rhodamine B discoloration activity upon light irradiation. Under UV irradiation, the electrically conductive MXene in combination with *in situ* formed semiconducting titanium oxide induces photogenerated charge carriers, which could potentially be used in photocatalysis. On the other hand, due to film transparency, white light irradiation can bleach the adsorbed dye *via* photolysis. This study opens the door for using MXene thin films as multifunctional coatings with conductive and potentially self-cleaning properties.

## 1 Introduction

Thin film processing holds promise for the development of advanced electronic devices.^1^ However, conventional transparent conductive thin films often lack effective self-cleaning properties,^2,3^ while some applications may benefit from the combination of high electrical conductivity and photocatalytic activity: for example, self-cleaning solar panels. On the other hand, most excellent self-cleaning thin films tend to exhibit high resistivity and poor optical transparency.^2^ For instance, P-doped TiO_2_ shows a high resistivity of about 1–6 Ω cm and an optical transmittance below 80% in an ∼450 nm thick film.^4^ Thus, achieving thin films with high electrical conductivity, combined with photocatalytic activity and high optical transparency, is a challenge. Some of these requirements can be satisfied with MXenes due to their exceptional physicochemical properties.^5−7^

MXenes are a large family of 2D materials consisting of transition metal carbides, nitrides, and carbonitrides, first described in 2011.^8^ MXene films exhibit high conductivity (>20000 S cm^–1^ according to some reports),^9^ hydrophilicity, and excellent water dispersibility,^10^ and robust photon absorption in a wide wavelength range.^11^ To date, Ti_3_C_2_T_*x*_ MXene has received more attention than other MXenes due to its ease of fabrication, excellent electrochemical performance, tunable optical properties, and fair stability.^12,13^ Solid solution MXenes, such as Ti_3_CNT_*x*_, are also starting to gain attention due to their intriguing physical properties.^14^ Density functional theory (DFT) studies predict a higher density of states at the Fermi level in Ti_3_CNT_*x*_, resulting in electrical conductivity better than that of Ti_3_C_2_T_*x*_.^15^ Similar to other MXenes, Ti_3_CNT_*x*_ hydrophilic surface functional groups make it an excellent candidate for water-based thin film processing, including layer-by-layer (LbL) assembly.^16^

Several researchers demonstrated optoelectronic applications of Ti_3_CNT_*x*_. For instance, a fast carrier relaxation in few-layered Ti_3_CNT_*x*_ nanosheets may potentially enable applications as ultrafast optical modulation devices.^17^ Ti_3_CNT_*x*_ also shows better electrocatalytic activity in hydrogen evolution reaction than Ti_3_C_2_T_*x*_.^18^ In another study, the photocatalytic ability of Ti_3_CNT_*x*_/TiO_2_ combined with ruthenium has been demonstrated,^19^ where a combination of Ti_3_CNT_*x*_, TiO_2_, and Ru outperformed conventional P25 photocatalyst for CO_2_ reduction.

Despite the promise of MXenes in optoelectronic applications, Ti_3_CNT_*x*_ has poor environmental stability. In many applications, chemical stability is a common problem for MXenes due to their reactivity toward oxidizers (e.g., O_2_) and water under ambient conditions,^20−22^ in contrast to their counterpart bulk transition-metal carbides and nitrides, which are stable (beyond a few atomic layers).^23^ Based on the comparison of experimental activation energies of hydrolysis of Ti_2_C, Ti_3_C_2_, Ti_3_CN, and Nb_2_C, Ti_3_CN has the lowest activation energy and stability compared to other MXenes.^20^ It is commonly believed that this is due to the crystal structure of Ti_3_CNT_*x*_, in which nitrogen atoms are randomly substituted by carbon atoms in the X element sites. Thus, either nitrogen or carbon atoms act as substitutional defects in the structure of another, leading to enhanced chemical reactivity of Ti_3_CNT_*x*_.^20,24^ This also explains experimentally measured higher rates of Ti_3_CNT_*x*_ hydrolysis compared to its carbide cousin MXene with a similar monolayer thickness and composition (Ti_3_C_2_T_*x*_).^20^ Therefore, increasing the chemical stability of Ti_3_CNT_*x*_ has become a matter of interest.

MXenes can be dispersed in nonaqueous solvents, such as alcohols etc. to avoid the hydrolysis.^21^ This led to recent attempts to suppress the degradation of Ti_3_CNT_*x*_ by exchanging water with organic solvents.^25^ Ti_3_CNT_*x*_ was stable for up to 7 days when dispersed in ethanol and *N*,*N*-dimethylformamide (DMF), although the solubility of oxygen in both organic solvents many times exceeds the oxygen solubility in water under ambient conditions. However, the organic-based dispersions of MXenes often have poor colloidal stability at high concentrations and are less environmentally friendly.^26^

Apart from the oxidation and hydrolysis of MXenes in dispersions, mitigating oxidation issues in thin-film form is crucial to ensure the stable performance of optoelectronic devices during the operation. This concern is particularly pronounced in Ti_3_CNT_*x*_ thin film. In a previous study, the sheet resistance of Ti_3_CNT_*x*_ thin films increased 3.5 times after 30 h of storage under ambient conditions, while Ti_3_C_2_T_*x*_ exhibited only a 10% sheet resistance change.^27^ Thus, thin-film fabrication from a freshly made water-based solution and further stabilization of MXene in the solid state could facilitate optoelectronic applications.

For a more widely studied Ti_3_C_2_T_*x*_ MXene, several approaches were developed to improve its stability in a thin film. For example, high-temperature hydrogen annealing at 900 °C yielded Ti_3_C_2_T_*x*_ with high environmental stability.^28^ However, this approach can be problematic if applied to Ti_3_CNT_*x*_ thin film, since it quickly decomposes at temperatures over 150 °C.^27^ The stability of Ti_3_C_2_T_*x*_ was also improved through the application of a perfluorosulfonic acid coating,^29^ which hinders access to oxygen and water from air. However, the barrier layer coating may also decrease the electron transport efficiency in the Ti_3_CNT_*x*_ films.

Another promising approach utilizes the barrier properties of graphene^30^ and reduced graphene oxide (rGO). The thin rGO film shows low gas permeability when exposed to air,^31^ and it has been used to protect other environmentally sensitive materials such as Ag nanowires and perovskites.^32−34^ Because the rGO is a derivative of 2D graphene oxide (GO), initial interactions of the material with GO can facilitate intimate interfacial bonding with the T_*x*_ groups of Ti_3_CNT_*x*_ MXene. Recently, the possibility of obtaining rGO from graphene oxide (GO) at a low temperature (150 °C) with l-ascorbic acid as a reducing agent has been demonstrated.^35^ In addition, it can protect MXene from oxidation during processing.^13^ Therefore, the reduction of GO to rGO with l-ascorbic acid could be applied as the next step, strengthening the connection between rGO and MXene.

In this study, we aimed to fabricate rGO/Ti_3_CNT_*x*_ thin films under ambient conditions using a facile spin-coating method to combine two, usually incompatible, properties in one material: good electrical conductivity of MXene and a pronounced band gap originating from surface titanium oxide and rGO. A similar thin-film material based on controllably oxidized Ti_3_C_2_T_*x*_, combining high electrical conductivity due to MXene and photoresponse due to tightly integrated *in situ* TiO_2_, has been reported before.^36^

The underlying mechanism relies upon Ti_3_CNT_*x*_ MXene atomically thin core layers providing conductivity while subjecting surface layers to mild oxidation to create a band gap. The extent of oxidation is small enough not to interfere with the high conductivity of the MXene core. The excellent stabilization of MXene optoelectronic properties by rGO was further evidenced by increased electrical conductivity and more efficient photoinduced charge carrier separation while maintaining high performance stability at room temperature during 21 days of testing. Interestingly, even after 7 months of storage, rGO/Ti_3_CNT_*x*_ still maintained its electrical conductivity. In addition, the thin film exhibited a high optical transparency of 74% in the visible region. To demonstrate the self-cleaning potential of our rGO/Ti_3_CNT_*x*_ thin films, we tested their performance toward discoloration of solid-state rhodamine B under either ultraviolet or white-light irradiation. We have found it difficult to disentangle the contributions of photocatalysis and photolysis. Nevertheless, our findings may pave the way for Ti_3_CNT_*x*_ MXene to be used in multifunctional thin-film coatings for optoelectronic and self-cleaning solar cell applications.

## 2 Experimental Section

### 2.1 Materials

Hydrochloric acid (HCl, 37 wt %) was obtained from Merck. Aqueous solutions of tetramethylammonium hydroxide (TMAOH, 25 wt %), sulfuric acid (H_2_SO_4_, 98 wt %), and phosphoric acid (H_3_PO_4_, 85 wt %), as well as powders of potassium permanganate (KMnO_4_), and l-ascorbic acid (LAA, 99% purity) were acquired from Sigma-Aldrich. Lithium fluoride (LiF, 97% purity) powder was purchased from Acros Organics, and solid rhodamine B (C_28_H_31_ClN_2_O_3_) was obtained from Glentham life science. Isopropanol and acetone (99.5% purity) were obtained from Warchem. The 15 × 15 mm square glass substrates for thin-film coating were purchased from ChemLand. Double-distilled water (DDW) was used in this study.

### 2.2 Preparation of Ti_3_AlCN MAX Phases

To prepare Ti_3_AlCN, titanium (Ti, 99.99 wt % purity, < 44 μm, Alfa Aesar, USA), aluminum nitride (AlN, N-32.0% min, < 44 μm, Alfa Aesar, USA), and graphite (C, 99.9995 wt % purity, < 44 μm, Alfa Aesar, USA) were mixed to 25 g total in a molar ratio of Ti:AlN:C = 3:1:1, using a Turbula T2F mixer. The powders were mixed in a 125 mL high-density polyethylene (HDPE) bottle with ten 10 mm yttria-stabilized zirconia (YSZ) balls for 3 h at ∼56 rpm. The mixed powders were transferred into an alumina crucible and placed in a tube furnace with a continuous argon flow. The furnace was heated (10 °C/min) from room temperature to 1500 °C and held at that temperature for 105 min. After cooling to room temperature, the starting powder turned into a brick-like block of Ti_3_AlCN MAX phase. This block was crushed into powder using a jaw crusher (MTI KJ group, China) and sieved to obtain particle sizes of less than 44 μm for further steps.

### 2.3 Synthesis of Ti_3_CNT_*x*_ MXene Nanoflakes

Ti_3_CNT_*x*_ MXene was synthesized by selectively etching the Ti_3_AlCN MAX phase *via* a microwave-assisted hydrothermal method. First, 300 mg of solid LiF was mixed with 15 mL of aqueous HCl (6 M) and stirred for 10 min. 250 mg of Ti_3_AlCN powder was slowly added to the LiF/HCl solution, and the mixture was sonicated for 1 min. Next, the mixture was transferred to a microwave reactor (Magnum II Microwave reactor, ERTEC, Poland). The microwave reactor was programmed with an MW irradiation power of 480 W and a reaction time of 4 h at 150 °C. The microwave radiation can penetrate and heat the entire volume of the absorbing medium (water) at the same time, avoiding temperature gradients and resulting in uniform and fast heating.

After the etching process, the sedimented MXene flakes were washed several times with DDW until the pH reached values of ∼ 6. At this stage, multilayered Ti_3_CNT_*x*_ MXene flakes were obtained. The flakes were delaminated by adding a tetramethylammonium hydroxide (TMAOH) solution in a ratio of 5 mL per 1 g of MXene and stirring for 24 h at room temperature. Next, the mixture was continuously washed with DDW (9000 rpm and 5 min per washing cycle) until a pH of ∼ 6. Furthermore, the MXene was resuspended in DDW and centrifuged at 3500 rpm for 5 min to collect the black supernatant consisting of single-layered (SL) MXene with some amount of a few-layered MXene, while the sediment containing multilayered flakes was discarded.

### 2.4 Synthesis of Graphene Oxide

Graphene oxide (GO) was synthesized using a modified Hummer’s method. Graphite flakes (5 g) were added to 110 mL of 98 wt % H_2_SO_4_ and 11.5 mL of 85 wt % H_3_PO_4_. The mixture was then stirred with a mechanical stirrer for 1 h at 180 rpm. In the next step, 15 g of KMnO_4_ was added gradually to the reaction mixture with cooling by an ice/water bath, maintaining the temperature at 5 °C. The mixture was then stirred for 24 h at room temperature. Finally, it was washed with DDW and centrifuged until a pH of ∼ 6.

### 2.5 Preparation of Ti_3_CNT_*x*_ MXene Thin Films

Ti_3_CNT_*x*_ thin films were deposited on glass by using a spin-coating technique. The 15 × 15 mm glass substrates were bath sonicated in acetone, isopropanol, and DDW for 5 min each. After that, the substrates were dried on a hot plate at 150 °C for 3 min. Before coating, the substrates were treated with UV–O_3_ for 1 h to improve the hydrophilicity of the surfaces. The distance between the substrate and the UV lamp was set at 2.5 cm. An aqueous dispersion (2.38 mg mL^–1^) of SL Ti_3_CNT_*x*_ was used for spin-coating. For this, 60 μL of the solution was uniformly dispensed on the substrate and the substrate was subsequently rotated at 2000 rpm for 30 s. Films of different thicknesses can be obtained by varying the number of spin-coating cycles (3, 5, 7, and 9). The prepared SL Ti_3_CNT_*x*_ thin films on glass were stored in a desiccator filled with argon to prevent degradation.

### 2.6 Preparation of rGO/Ti_3_CNT_*x*_ Heterostructured Thin Films

**Figure 1:**
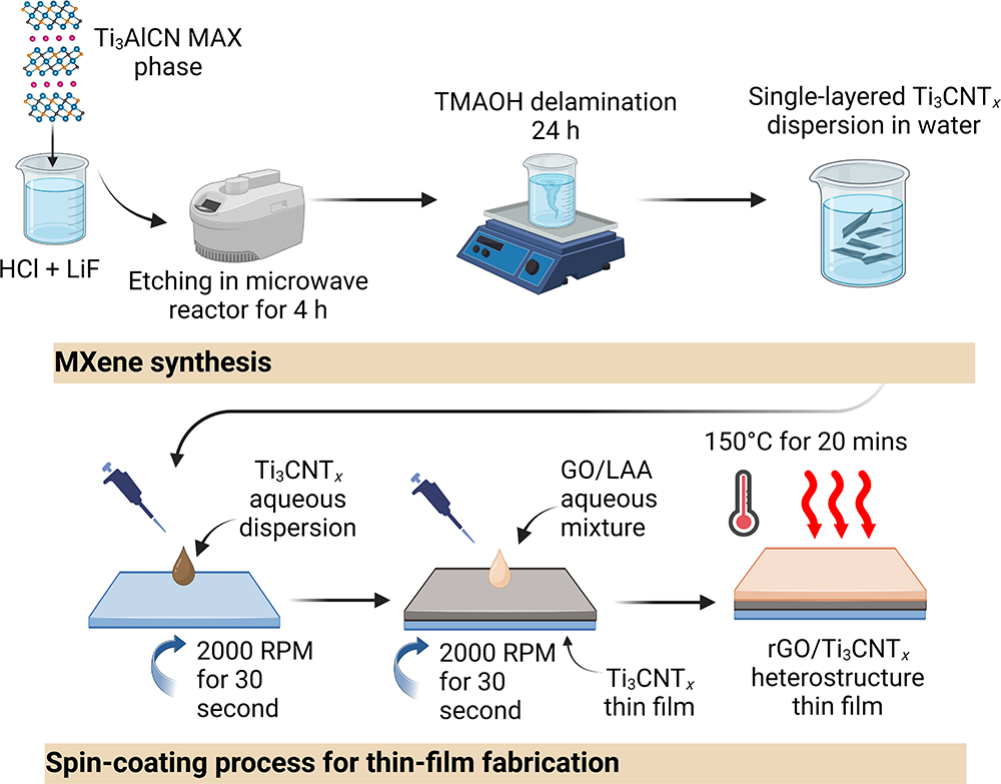
Schematic diagram of Ti_3_CNT_*x*_ MXene synthesis and subsequent thin film fabrication. Created with BioRender.com.

First, an aqueous dispersion of GO (4 mg/mL) was vortexed (5 min) with l-ascorbic acid (LAA) solution (50 mM) in a 1:1 volume ratio. Afterward, the GO/LAA solution (120 μL) was uniformly dispensed onto previously prepared Ti_3_CNT_*x*_ thin films and spin-coated at 2000 rpm for 30 s. The previous study demonstrated that LAA is a safe, cost-effective, and efficient reducing agent.^35^ After the spinning, the sample was placed on a hot plate at 150 °C for 20 min to complete the *in situ* reduction process. In the end, we obtained the heterostructured rGO/Ti_3_CNT_*x*_ film. The synthesis process of Ti_3_CNT_*x*_ MXene and the fabrication of the rGO/Ti_3_CNT_*x*_ thin film are schematically shown in Figure 1. In this study, the optimized rGO/Ti_3_CNT_*x*_ thin film was made by 7 coating cycles of Ti_3_CN and 1 coating cycle of the protective rGO layer.

### 2.7 Characterization

The morphology of Ti_3_CNT_*x*_ was studied using scanning electron microscopy (SEM Hitachi SU3500), field emission scanning electron microscopy (FE-SEM, Hitachi S5500, Hitachi, Tokyo, Japan), and transmission electron microscopy (TEM, Tecnai G2, Eindhoven, Netherlands). The thin film samples for SEM imaging were prepared on Si substrates. Energy-dispersive X-ray spectroscopy (EDS) was used to study the elemental composition of the samples. X-ray diffraction patterns (XRD) were measured with a Bruker D8 Advanced (Billerica, MA).

The zeta potential of Ti_3_CNT_*x*_ flakes in dispersion was characterized by a Zetasizer NANO ZS ZEN3500 (Malvern Instruments, Malvern, UK) with a detector collecting scattered light at 173°. The reported results are averages from 10 repeated measurements. The chemical composition of the Ti_3_CNT_*x*_ and rGO/Ti_3_CNT_*x*_ heterostructure was studied using attenuated total reflectance Fourier transform infrared (ATR-FTIR) spectroscopy (Nicolet iS5, Thermo Electron, Waltham, MA).

Raman spectra of the Ti_3_AlCN MAX phase and SL Ti_3_CNT_*x*_ MXene flakes were acquired by a Renishaw InVia confocal Raman microspectrometer with 532 nm excitation laser and 1200 mm^–1^ grating. For each measurement, we used 30 s acquisition time, 3 accumulations, and laser powers of 1% and 0.5% for the Ti_3_AlCN MAX phase and SL Ti_3_CNT_*x*_ MXene, respectively, to prevent their laser-induced damage.

The optical properties of thin films were measured by a double-beam UV–visible (UV–vis) spectrometer (Evolution 220, Thermo Scientific) with an integration time of 0.3 s, a wavelength resolution of 1 nm, and a scanning speed of 200 nm min^–1^. The blank glass substrate was used as a reference in the spectral acquisition.

The electrical properties of the films were measured with a digital multimeter (Keithley DAQ6510, USA). A two-point probe with a 1 cm gap between the contacts was used to measure the sheet resistance of the films. The measurements were taken at four different spots, and the reported sheet resistance results were obtained by taking the average of these values. The environmental stability of Ti_3_CNT_*x*_ and rGO/Ti_3_CNT_*x*_ thin films was assessed by measuring the change in the sheet resistance (*R*/*R*_0_) of the samples over time (*R* is the sheet resistance at a specific time, and *R*_0_ is the initial sheet resistance value). The films were stored at ambient temperature and exposed to air for 21 days. In addition, the current–voltage curve was measured by using a sourcemeter unit (Keithley SMU 2450, USA).

The optoelectronic properties of the thin films were assessed by studying the photocurrent density in the samples. Here, the films were placed in a photochemical reactor (PhotoCube Photochemical reactor, ThalesNano, Budapest, Hungary) and irradiated with either white (400–700 nm, luminous flux of 5920 lm) or UV (365 nm, radiant flux of 44.8 W) light with five alternating light on-and-off cycles (each cycle was carried out for 60 s). The current was recorded by a digital multimeter (Keithley DAQ 6510, USA) with a bias voltage of 1.5 V.

### 2.8 Discoloration of Rhodamine B Dye

Discoloration experiments were performed in the solid state. 400 μL of Rhodamine B (RhB) in DDW (70 mg/L) was drop-cast on either a Ti_3_CNT_*x*_ or rGO/Ti_3_CNT_*x*_ thin film. The samples were left overnight to ensure the dye was adsorbed and dried on the films. To monitor RhB photolysis, samples were prepared in a similar way on a bare glass substrate without the thin film coating. The discoloration was performed in a photoreactor using either UV (365 nm, radiant flux of 44.8 W) or white (400–700 nm, luminous flux of 5920 lm) light. The color changes of the films were measured by UV–vis spectroscopy (Evolution 220, Thermo Scientific) at various illumination times. The UV–vis absorption was measured at five random spots of the films using an in-house-made sample holder, and the results were presented as the mean value and standard deviation. The RhB discoloration is determined by comparing the dye absorbances before (*A*_0_) and after (*A*) exposure to light. Baseline correction was applied to each of the measured absorption spectra to remove the contribution from the thin films.

## 3 Results and Discussion

Ti_3_CNT_*x*_ MXene holds promise for optoelectronic applications due to its high absorption of electromagnetic waves, moderate conductivity, and broad-band saturable absorption.^14,17^ However, Ti_3_CNT_*x*_ is prone to degradation under ambient conditions, and its optoelectronic characteristics have not yet been fully explored. To minimize the MXene degradation, we develop the optimal formulation of the rGO/Ti_3_CNT_*x*_ heterostructured thin film, which is conductive and highly stable, and investigate its self-cleaning performance. The thin film was fabricated by an all-solution process under ambient conditions.

First, graphene oxide (GO) solution was prepared using a modified Hummer’s method. The sheet-like appearance of GO with a typical folded and wrinkled structure observed with SEM is presented in Figure S1 in the Supporting Information. GO UV–vis spectra (Figure S2a) agree with previously reported results, with a distinctive sharp peak at ∼232 nm and a broad shoulder at ∼293 nm.^37^ ATR-FTIR spectra (Figure S2b) reveal the presence of various functional groups on GO (O–H at 3371 cm^–1^, C=O at 1718 cm^–1^), and C=C bonds (1622 cm^–1^). Raman spectra of the as-prepared GO show the G band at ∼1590 cm^–1^ and the D band at ∼1350 cm^–1^ (Figure S3).

**Figure 2:**
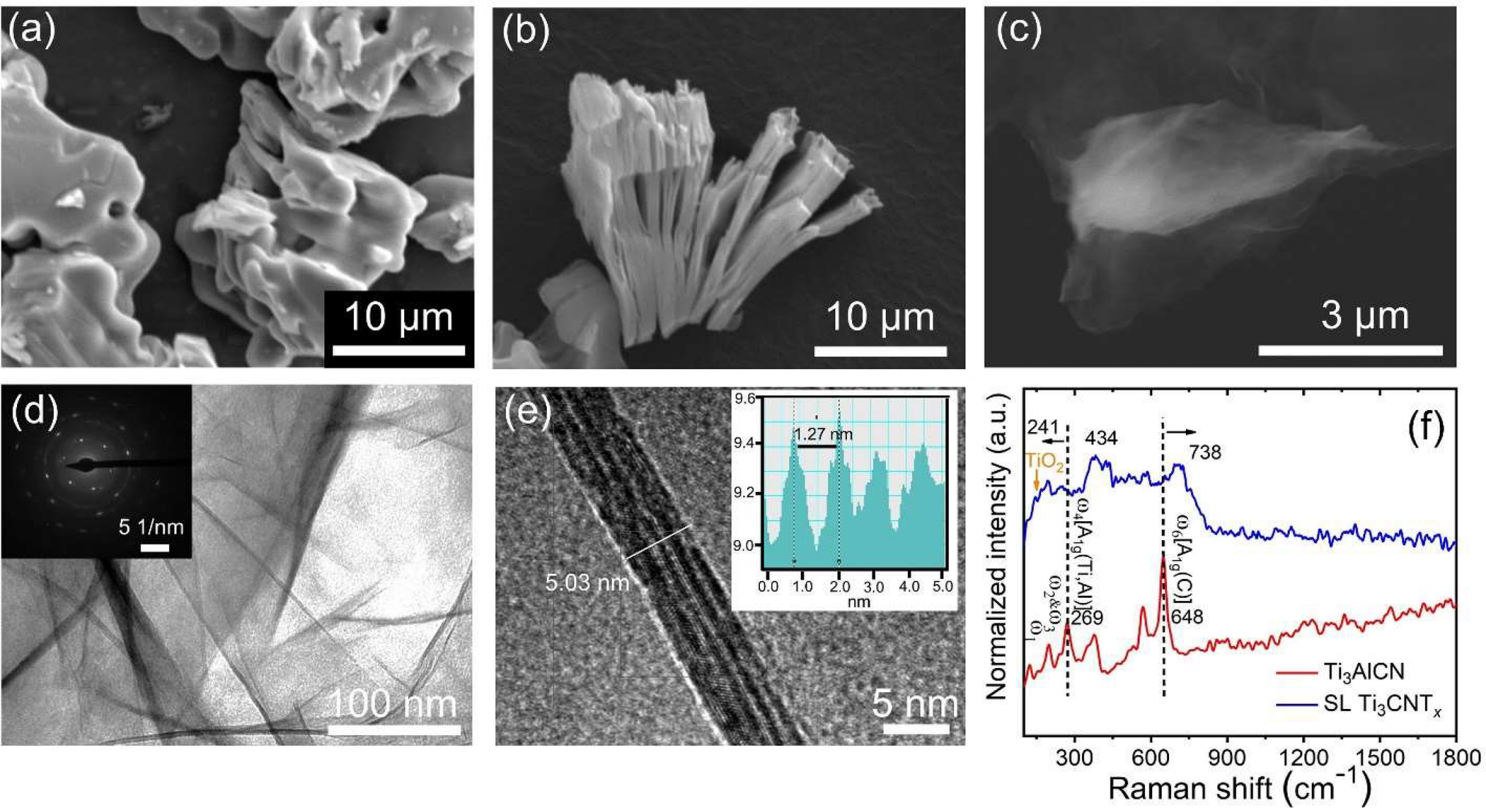
SEM images of (a) parent Ti_3_AlCN MAX phase and (b) accordion-like multilayered (ML) Ti_3_CNT_*x*_ MXene obtained after microwave-assisted hydrothermal reaction for 4 h. (c) SEM and (d) TEM images of single-/few-layered (SL) Ti_3_CNT_*x*_ after delamination using TMAOH. (e) Cross-sectional TEM image of SL Ti_3_CNT_*x*_. (f) Raman spectra of Ti_3_AlCN MAX phase and SL Ti_3_CNT_*x*_. The orange arrow denotes contribution from TiO_2_ anatase. The inset of (d) shows the selected area diffraction pattern (SADP) of SL Ti_3_CNT_*x*_. The inset of (e) shows the intensity pattern extracted from the inverse fast Fourier transform of the TEM image.

Next, we produced Ti_3_CNT_*x*_ from the Ti_3_AlCN MAX phase (Figure 2a) *via* a microwave-assisted hydrothermal method. The combination of hydrothermal treatment and microwave heating reduces the reaction time from 24 h (conventional etching) to 4 h, while maintaining decent material quality. After 4 h of the microwave-assisted hydrothermal reaction, we successfully etched out Al layers and obtained a multilayered accordion-like Ti_3_CNT_*x*_ structure, as demonstrated in an SEM image (Figure 2b). Next, we prepared single-layered (SL) Ti_3_CNT_*x*_ nanoflakes *via* TMAOH delamination and used the obtained aqueous colloidal dispersion as a coating ink. After the delamination, we obtained ultrathin few- to single-layered MXene flakes (Figure 2c). In this regard, TMAOH could effectively weaken the interaction between M–X layers by increasing the interflake distance, allowing us to obtain SL Ti_3_CNT_*x*_. Besides acting as an intercalating agent, TMAOH treatment also removes etching byproducts such as AlF_*x*_ and Al(OF)_*x*_,^38^ which are present on the surface of MXene, further stabilizing Ti_3_CNT_*x*_ layers.

We further confirmed the single- and few-layered structures of SL Ti_3_CNT_*x*_ nanoflakes by transmission electron microscopy (TEM). The TEM image (Figure 2d) showed the transparent Ti_3_CNT_*x*_ nanoflakes free from apparent pinholes and cracks, indicating the high quality of synthesized few- to single-layered nanosheets. The selected area diffraction pattern (SADP) confirmed the high crystallinity of SL Ti_3_CNT_*x*_ (inset of Figure 2d). Based on the cross-sectional high-resolution TEM image (Figure 2e), we found that the interlayer spacing of SL Ti_3_CNT_*x*_ is 1.27 nm (inset of Figure 2e).

Furthermore, we confirmed the delamination process by XRD (Figure S4), where the (002) MXene peak is located at a 2θ value of 4.3°. The diffraction peaks of the parent MAX phase vanished completely, indicating successful MXene preparation.^22^ EDS analysis revealed that our MXene is mainly composed of titanium, carbon, and nitrogen (Figure S5). The aluminum content was reduced to a residual amount. Atomic and weight percentages of elements identified by EDS are presented in Table S1 in the Supporting Information. Fluorine and oxygen (see full EDS mapping of SL Ti_3_CNT_*x*_ in Figure S6) may be associated with surface functional groups of the MXene.^39^

Raman spectroscopy is a powerful tool to characterize materials and study their chemical transformations. It was used to confirm the successful etching of MXene from its parent MAX phase.^40^Figure 2f shows Raman spectra of our Ti_3_AlCN and delaminated SL Ti_3_CNT_*x*_. Ti_3_AlCN exhibits narrow (as expected for a bulk crystalline material) vibration modes in the range of 100–800 cm^–1^. The most pronounced peaks belong to A_1g_(Ti,Al) and A_1g_(C), assigned to ω_4_ (269 cm^–1^) and ω_6_ (648 cm^–1^), respectively. We also observe ω_1_, ω_2_, and ω_3_ modes in the Raman spectra. Altogether, ω_1_, ω_2_, ω_3_, and ω_4_ modes can be assigned to vibrations involving Al.^41^ We assigned the ω_6_ mode to X atom sublattice vibrations.^41^ In particular, based on the relationship between the phonon energy and the reduced mass,^42^ a vibrational peak located at ∼569 cm^–1^ might be related to N atoms, as Ti_3_AlCN contains carbon and nitrogen at X sites.

After etching, the ω_1_, ω_2_, and ω_3_ modes disappear, implying the removal of Al atoms. Moreover, the ω_4_ mode is shifted from 269 to 241 cm^–1^. Shifting to a lower wavenumber relates to the vibration involving C and functional groups.^40^ On the other hand, the ω_6_ mode is shifted from 648 to 738 cm^–1^. Shifting to a higher wavenumber was observed previously.^40^ In addition, the pronounced 434 cm^–1^ peak comes from a contribution of surface functional groups bonded to titanium atoms and their in-plane vibrations.^40^ Furthermore, we observe a weak peak at ∼142 cm^–1^ from the TiO_2_ anatase present in SL Ti_3_CNT_*x*_ MXene. The small amount of TiO_2_ indicates the ongoing MXene degradation observed even in freshly made MXene samples, as Ti_3_CNT_*x*_ shows low chemical stability. We harnessed this superficial TiO_2_ to impart UV sensitivity to Ti_3_CNT_*x*_ films, thereby facilitating their self-cleaning activity.

We confirmed the colloidal stability of our SL Ti_3_CNT_*x*_ by zeta potential measurements (Figure S7) and UV–vis absorption spectra (Figure S8). Ti_3_CNT_*x*_ MXene shows a negative zeta potential of −22.13 ± 0.37 mV. The UV–vis absorption spectra of the SL Ti_3_CNT_*x*_ nanoflake solution show an inflection point at 667 nm, as reported previously.^27^ This weak plasmonic peak contrasts with the clear plasmonic peak typically observed for other MXenes, such as Ti_3_C_2_T_*x*_ (∼800 nm) or Ti_2_CT_*x*_ (∼550 nm).^43^ Overall, these results are in good agreement with those previously reported for Ti_3_CNT_*x*_ MXene delaminated *via* conventional HF/MILD and LiF/HCl methods.^27,43^

We calculated the mass extinction coefficient of our MXene from UV–vis data by calibrating the UV–vis absorption spectra for the 667 nm peak maximum (Figure S9). The obtained value is 23.49 L g^–1^ cm^–1^, slightly lower than previously reported for Ti_3_CNT_*x*_ produced with HF/TMAOH (27.0 L g^–1^ cm^–1^) or LiF/HCl (27.6 L g^–1^ cm^–1^) etching.^27^ These differences can be attributed to variations in flake size and surface functional groups.^44^ In general, the contribution of processes beyond absorbance (*e.g.*, scattering and reflection of light) cannot be excluded in the UV–vis spectra of colloidal particles.

**Figure 3:**
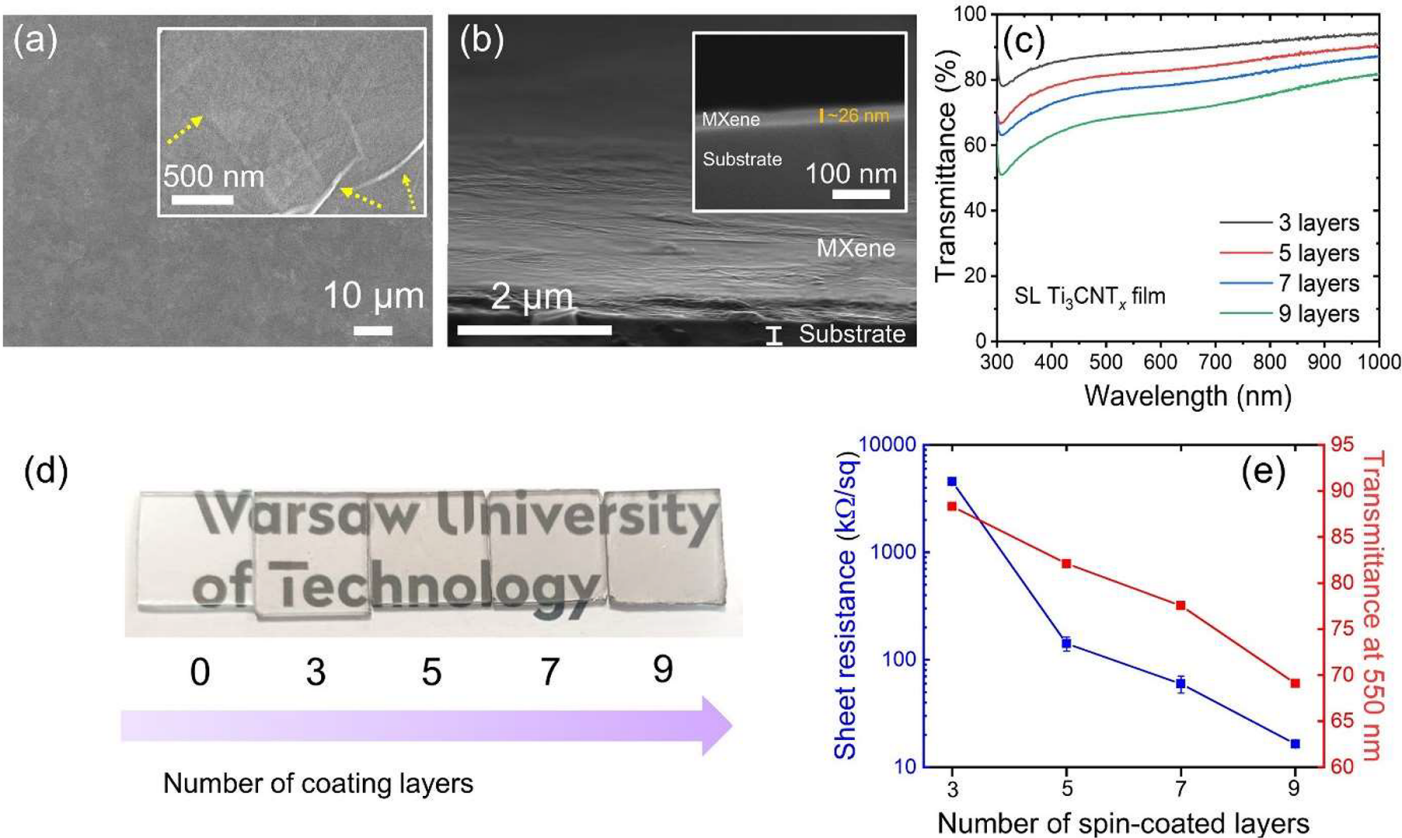
(a) Smooth surface morphology of Ti_3_CNT_*x*_ film on a Si substrate. (b) Tilted cross-sectional image of Ti_3_CNT_*x*_ thin film on the substrate. (c) UV–vis spectra of Ti_3_CNT_*x*_ thin films produced by varying the number of spin-coating layers. (d) Digital photographs of Ti_3_CNT_*x*_ thin films deposited on glass substrates. (e) Sheet resistance and optical transmittance at 550 nm of Ti_3_CNT_*x*_ with a varied number of layers. The inset of (a) shows the high-magnification image of Ti_3_CNT_*x*_ film with yellow arrows marking the interconnected Ti_3_CNT_*x*_ flake’s edge. The inset of (b) shows the high magnification of the cross-sectional image of the Ti_3_CNT_*x*_ thin film.

The hydrophilic SL Ti_3_CNT_*x*_ flakes could be easily coated on a glass substrate treated with UV light/O_3_ to improve hydrophilicity. A colloidal solution of Ti_3_CNT_*x*_ MXene was drop-cast and spin-coated under ambient atmosphere according to the procedure in Figure 1. The surface of air-dried thin films was smooth, as shown in Figure 3a, without apparent pores, indicating tight contacts between the flakes (inset in Figure 3a) and their uniform in-plane orientation parallel to the substrate surface. The tilted cross-sectional image in Figure 3b confirms that the flakes adhere well to the substrate surface, forming a film with a thickness of ∼26 nm (inset of Figure 3b).

We were able to change the transparency of the film by varying the number of spin-coating cycles. We performed the layer-by-layer (LbL) spin-coating, increasing the number of Ti_3_CNT_*x*_ layers from 3 to 9. The transmittances of these samples are shown in Figure 3c. The transmittance values of thin films at 550 nm are 88, 82, 78, and 69% for 3, 5, 7, and 9 coating cycles, respectively. Variation of the thin-film transparency is also illustrated by digital photographs of the samples in Figure 3d. The samples become darker with more Ti_3_CNT_*x*_ layers applied in repeated spin-coating cycles. However, the film remains sufficiently transparent (over 69%) even after 9 cycles. The high transparency originates from Ti_3_CNT_*x*_ not exhibiting an extinction peak in the visible region, oppositely to Ti_3_C_2_T_*x*_ and Ti_2_CT_*x*_.^5^ Therefore, the Ti_3_CNT_*x*_ thin film has potential as a transparent conducting coating.

We further analyzed the relationship between the optical and electrical properties of Ti_3_CNT_*x*_ thin films (Figure 3e). We expected a tradeoff between the thin film sheet resistance and optical transmittance. Strikingly, while the transmittance of Ti_3_CNT_*x*_ thin films at 550 nm only varies by 19% between the extreme samples, the sheet resistance ranges between 4.56 and 16 kΩ/sq. Moreover, we calculated the optoelectronic figure of merit (FoM) of the Ti_3_CNT_*x*_ thin film (Figure S10). The calculated FoM is 0.09, close to the previously reported value.^27^ The previous experimental study showed that Ti_3_CNT_*x*_ has a lower conductivity than Ti_3_C_2_T_*x*_ owing to a random distribution of N atoms at the X sites and the presence of atomic defects, which may act as electron scattering centers.^27^ The conductivity may further be reduced due to the MXene structure degradation, as discussed in Raman analysis. Therefore, developing an ambient-temperature thin-film processing method is an important step forward for MXene applications. Although Ti_3_CNT_*x*_ thin films show a lower conductivity than Ti_3_C_2_T_*x*_, it is still superior to other solution-processed conductive self-cleaning thin films and is comparable to those obtained by chemical vapor deposition (Table S2).

Previous studies revealed that Ti_3_CNT_*x*_ is less stable than Ti_3_C_2_T_*x*_, and the stability is even worse when fabricated as a thin film.^27^ It is known that MXene thin films degrade when exposed to a humid environment, which hampers many of their potential applications.^11^ Thus, we tested the environmental stability of Ti_3_CNT_*x*_ thin films with an increased number of spin-coating cycles. The tests were conducted by measuring the conductivity of the films exposed to ambient conditions for 7 days (Figure S11). Film resistance rapidly increased over time for the thinner films, with the 9-layer Ti_3_CNT_*x*_ film showing the best stability and the 3-layer film the worst. Consequently, thinner Ti_3_CNT_*x*_ films have extremely low environmental stability due to the increased surface-to-volume ratio of the MXene flakes exposed to air.

For further studies, we selected the Ti_3_CNT_*x*_ thin film fabricated by 7 cycles of spin-coating, which has optimal environmental stability that could be further improved by covering it with an rGO protective layer. In addition, this may lead to enhanced optoelectronic properties, such as more efficient photogenerated charge transfer and increased conductivity. The surface of rGO/Ti_3_CNT_*x*_ thin films (Figure S12a) has a smooth and transparent appearance, with rGO flakes effectively covering Ti_3_CNT_*x*_ flakes. The cross-sectional SEM image (Figure S12b) revealed that rGO/Ti_3_CNT_*x*_ adhered well to the substrate, exhibiting a pore-free structure. Moreover, the high-resolution cross-sectional (Figure S12c) and backscattered electron images (Figure S12d) reveal distinct thin-film layers, confirming the overall good connection between the substrate, Ti_3_CNT_*x*_, and rGO. Scanning tunneling electron microscopy (Figure S13) further illustrates the interfacial connection between rGO and Ti_3_CNT_*x*_. The image shows that larger flakes of wrinkled rGO cover smaller flakes of Ti_3_CNT_*x*_.

**Figure 4:**
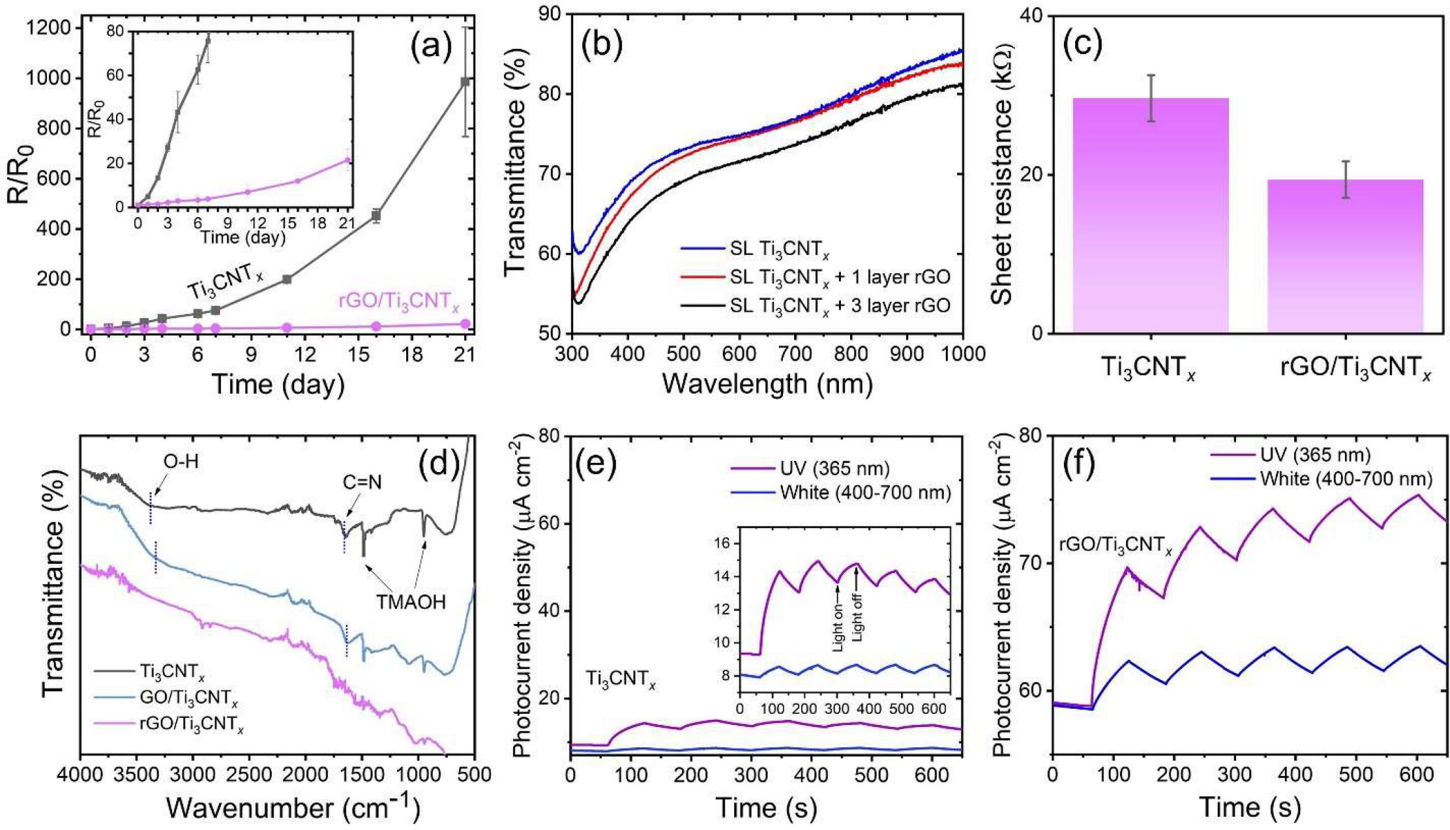
(a) Changes in relative resistance of thin films measured over 21 days of storage under an ambient atmosphere. (b) UV–vis transmittance spectra of Ti_3_CNT_*x*_ and rGO/Ti_3_CNT_*x*_ thin films. (c) Sheet resistance of Ti_3_CNT_*x*_, rGO/Ti_3_CNT_*x*_, and GO/Ti_3_CNT_*x*_ thin films. (d) FTIR spectra of Ti_3_CNT_*x*_ and rGO/Ti_3_CNT_*x*_ heterostructures. Photocurrent in (e) Ti_3_CNT_*x*_ and (f) rGO/Ti_3_CNT_*x*_ thin films under UV and white light irradiation at a bias voltage of 1.5 V. The measurements were performed at room temperature and in air. The insets of (a) and (e) show the magnified graphs.

The oxidation stability of the rGO/Ti_3_CNT_*x*_ and Ti_3_CNT_*x*_ thin films was assessed by exposing films to air at ambient temperature for 21 days. For comparison, we also prepared GO/Ti_3_CNT_*x*_ thin films. Figure 4a shows that the Ti_3_CNT_*x*_ film is unstable, with the *R*/*R*_0_ value reaching 986 after 21 days of the stability test. rGO/Ti_3_CNT_*x*_ thin films showed a 45-fold stability enhancement, with R/R_0_ reaching 22. The high stability of rGO/Ti_3_CNT_*x*_ heterostructure is due to the fact that rGO is more hydrophobic than MXenes and has a low gas and water permeability when exposed to ambient air.^31^ In contrast to rGO, the stability of the GO/Ti_3_CNT_*x*_ thin film is moderate with an *R*/*R*_0_ value of 296 (Figure S14), revealing that GO is less effective in protecting Ti_3_CNT_*x*_ from ambient air. It is known that the water permeability of hydrophilic GO films is similar to that of an open aperture.^45,46^ We additionally examined the stability of thin films by conducting electrical measurement after storing them in ambient atmosphere for 7 months. The current–voltage curve (Figure S15) revealed that rGO/Ti_3_CNT_*x*_ maintained its high conductivity, while the neat Ti_3_CNT film exhibited a significantly lower current, with 2 orders lower magnitude. This result indicates the good oxidation resistance of the rGO/Ti_3_CNT_*x*_ thin film, which is crucial for ensuring its long-term functionality.

Next, we analyzed the effect of the spin-coated rGO overlayer on the optical transparency of the Ti_3_CNT_*x*_ thin films. We observed slightly reduced optical transmittance of the rGO/Ti_3_CNT_*x*_ thin film compared to neat Ti_3_CNT_*x*_ (Figure 4b), but overall, adding a few rGO layers on the surface of Ti_3_CNT_*x*_ does not deteriorate the superb transparency of the Ti_3_CNT_*x*_ thin film. Adding more rGO layers makes the films less transparent to visible light. Therefore, to fabricate the rGO/Ti_3_CNT_*x*_ thin film, we decided to use only one layer of rGO added on top of 7 layers of Ti_3_CNT_*x*_. In this heterostructure design, we obtained an optical transmittance of 74% at 550 nm and excellent electrical conductivity (details in Table S2).

We further compare the electrical properties of rGO/Ti_3_CNT_*x*_ with those of the bare Ti_3_CNT_*x*_ thin film (Figure 4c). The rGO layer reduces the sheet resistance of the Ti_3_CNT_*x*_ thin film by 30%. In contrast, when GO is used as a coating layer, the sheet resistance of the thin film increases (Figure S16). The conductivity improvement upon coating with rGO can be attributed to the intimate interface between rGO and Ti_3_CNT_*x*_ facilitated by van der Waals (vdW) interactions, creating a tight connection between Ti_3_CNT_*x*_ and an additional pathway for charge carriers in rGO/Ti_3_CNT_*x*_ heterostructure, just like in the case of other conductive electrodes.^33,47^ Thus, we conclude that rGO/Ti_3_CNT_*x*_ is a promising candidate as a conductive thin film that maintains a high transparency, sufficient conductivity, and robust environmental stability.

We performed an ATR-FTIR analysis to understand better the chemical interactions between Ti_3_CNT_*x*_ and rGO in the rGO/Ti_3_CNT_*x*_ heterostructure (Figure 4d). First, we analyzed Ti_3_CNT_*x*_. The broad peak centered at 3376 cm^–1^ is attributed to hydroxyl group −OH, and the peak at 1649 cm^–1^ may correspond to C=N.^13,48^ The other peaks, located at 1485 and 952 cm^–1^, are attributed to residual TMAOH cations from the intercalation process, as reported previously.^49^ For GO/Ti_3_CNT_*x*_, the intensities of −OH and C=N peaks are decreased and shifted toward lower wavenumbers: 3325 and 1630 cm^–1^, respectively. This may indicate the formation of hydrogen bonds between the −OH groups of Ti_3_CNT_*x*_ and GO or the masking effect induced by GO.^50^ After the conversion of GO/Ti_3_CNT_*x*_ to rGO/Ti_3_CNT_*x*_, several peaks are diminished.

Figure 4e,f shows the photoinduced current in Ti_3_CNT_*x*_ and rGO/Ti_3_CNT_*x*_ thin films under UV and white light irradiation, respectively. Both films showed a higher response toward UV compared with white light. To confirm our finding, we performed photocurrent measurements with various wavelengths from 625 nm (red light) down to 365 nm (UV) (Figure S17). Ti_3_CNT_*x*_ thin films show negligible photoresponse in the 625–500 nm wavelength range. A considerable improvement in photocurrent is achieved when the light wavelength reaches 457 nm (2.71 eV), and the highest photocurrent corresponds to UV irradiation. We attribute the photocurrent generation under UV light to the presence of *in situ* TiO_2_ on the Ti_3_CNT_*x*_ thin-film surface, similar to the previous study with Ti_3_C_2_T_*x*_.^36^ The photocurrent generation under TiO_2_ band gap energy might be related to the presence of native defects.

The rGO/Ti_3_CNT_*x*_ film also shows a higher sensitivity to UV light than to white light (Figure 4f, details in Figure S18). Notably, the rGO/Ti_3_CNT_*x*_ thin film exhibits a much higher dark current compared to neat Ti_3_CNT_*x*_, which is consistent with a lower sheet resistance of rGO/Ti_3_CNT_*x*_. The current increments (defined as *I*_L_ – *I*_D_)^51,52^ of the Ti_3_CNT_*x*_ thin film under UV and white light are 5.06 and 0.62 μA, respectively. In the case of rGO/Ti_3_CNT_*x*_, the current increment is higher for both UV and white light irradiation, *i.e.*, 10.88 and 3.79 μA, respectively. We also observed an increased photocurrent slope, indicating a slow photocurrent rise and decay rate in rGO/Ti_3_CNT_*x*_. These results indicated that depositing the rGO layer on the Ti_3_CNT_*x*_ surface can help separate photogenerated charge carriers and prolong the charge carrier lifetime. The slow photocurrent rise and decay were also observed previously in a partially oxidized TiO_2_/Ti_3_C_2_T_*x*_ thin film.^36^ Additionally, the current increment in Ti_3_CNT_*x*_ and rGO/Ti_3_CNT_*x*_ upon white light irradiation is smaller compared to the UV light, indicating that the film is less sensitive toward white light.

To reveal the contributions of rGO and Ti_3_CNT_*x*_ to the optoelectronic properties of the rGO/Ti_3_CNT_*x*_ heterostructure, we measured the photocurrent of the rGO thin film alone under UV and white light irradiation (Figure S19). The photoresponse of rGO contrasts with that of Ti_3_CNT_*x*_. In the case of rGO, we observe a stronger photocurrent under white light than UV light. Measurements with monochromatic light of different wavelengths (Figure S20) show that at 625 nm rGO already showed a considerable photoresponse, consistent with its narrow band gap. From a previous report, the band gap of rGO chemically reduced by LAA can be tuned to the near-infrared region (800 nm).^53^ Considering the photoresponse of rGO and Ti_3_CNT_*x*_ separately, we conclude that both rGO and TiO_2_ on the MXene surface can generate electron and hole pairs upon irradiation with white and UV light, respectively.

We showed that Ti_3_CNT_*x*_ thin films demonstrate sufficient conductivity originating from the MXene core and photosensitivity due to the presence of surface TiO_2_. Therefore, we tested the self-cleaning ability of the films under UV or white light irradiation using discoloration of a model cationic dye, rhodamine B (RhB). The negative zeta potential of the flakes with functional groups (−F and −O terminations) on the surface helps MXene effectively adsorb cationic dyes, such as RhB.^54,55^ Additionally, we also tested the RhB adsorption on the bare rGO thin-film surface. In contrast to the MXene-based thin film, we found that bare rGO showed a poor interaction with RhB (Figure S21).

The self-cleaning ability of Ti_3_CNT_*x*_ and rGO/Ti_3_CNT_*x*_ thin films illustrated by the discoloration of RhB was monitored with UV–visible spectroscopy. First, we analyzed the discoloration of RhB under UV and white light irradiation due to photolysis, *i.e.*, in the absence of the catalyst. Figure S22 shows a digital photograph of the RhB discoloration drop-casted on glass substrates. RhB forms a circular spot on the glass upon drying, with a higher RhB concentration observed at the circle edge. During the irradiation, RhB underwent moderate self-discoloration under UV and white light. In particular, RhB discoloration under white light was more pronounced than that under UV irradiation, as RhB absorbs light efficiently around 560 nm.^56^

**Figure 5:**
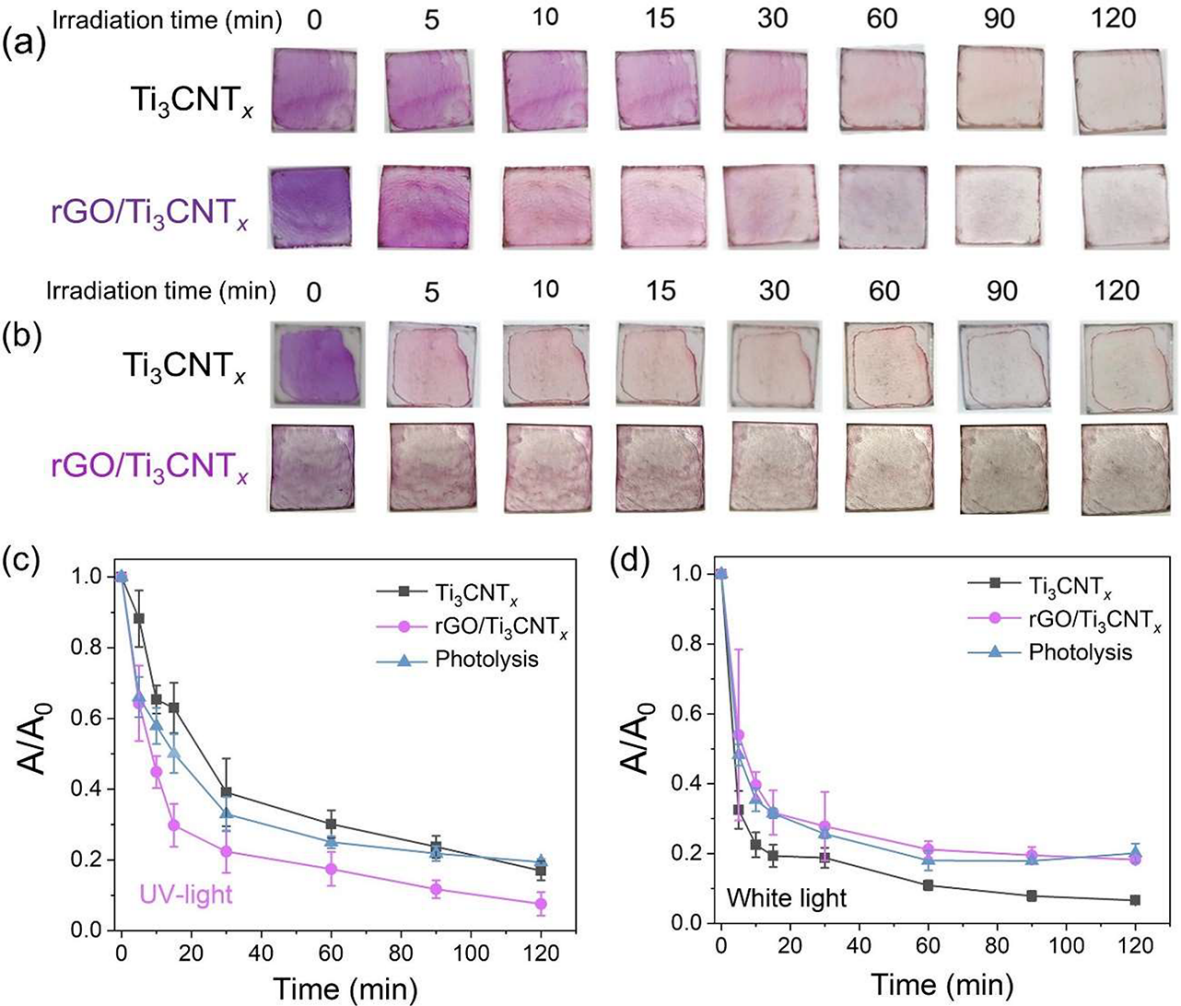
Digital photographs of rhodamine B discoloration on Ti_3_CNT_*x*_ and rGO/Ti_3_CNT_*x*_ thin films under (a) UV light and (b) white light irradiation. Rhodamine B discoloration on Ti_3_CNT_*x*_ and rGO/Ti_3_CNT_*x*_ thin films under (c) UV light and (d) white light irradiation. The irradiation was carried out for up to 120 min.

Figure 5a shows the visual progress of RhB discoloration on the film surface under UV irradiation. Ti_3_CNT_*x*_ and rGO/Ti_3_CNT_*x*_ thin films display excellent dye adsorption, consistent with their hydrophilicity.^57^ The color changes of RhB are observed under UV and white light irradiation for all of the films, as shown in Figure 5a,b, respectively. Figure 5c shows a decrease in the RhB absorbance peak under UV irradiation *versus* time. The rGO/Ti_3_CNT_*x*_ thin film shows more effective RhB discoloration than the Ti_3_CNT_*x*_ film. In this regard, the RhB discoloration performance under UV irradiation can be related to the photocurrent results in which rGO/Ti_3_CNT_*x*_ produces electron–hole pairs under UV more efficiently than Ti_3_CNT_*x*_.

However, the kinetics of photocatalytic dye degradation is almost similar to the kinetics of the dye photolysis, particularly under white light irradiation, which can be connected to the photosensitization effect of RhB (Figure 5d).^58^ The details of the RhB discoloration kinetics are shown in Figure S23. Although the photocurrent study suggests that rGO exhibited sensitivity toward white light, the contribution of the underlying photogenerated charge carriers is minimal in rGO/Ti_3_CNT_*x*_. This finding indicates that rGO acts as an electrically conducting channel (cocatalyst) in our system. We further evaluated the photostability of rGO/Ti_3_CNT_*x*_ by analyzing UV absorption spectra following 2 h exposure to UV irradiation (Figure S24). Remarkably, the thin film demonstrated excellent photostability without any noticeable changes in the absorption spectra.

As we mentioned previously, natively present TiO_2_ on the surface of Ti_3_CNT_*x*_ plays an important role in generating electron and hole pairs under UV irradiation. Therefore, the presence of this interfacial TiO_2_ must be taken into account to analyze the photogenerated charge transfer mechanism. We performed a Mott–Schottky analysis to determine the corresponding TiO_2_/Ti_3_CNT_*x*_ band edge position. From the Mott–Schottky plot (Figure S25), we obtained the flat band potential at −0.9 V vs Ag/AgCl (−0.70 V *vs* NHE). In addition, we observed a positive slope of the Mott–Schottky plot, indicating that the TiO_2_/Ti_3_CNT_*x*_ heterostructure is an n-type semiconductor. In n-type semiconductors, the flat band potential is ∼0.1 eV more positive than the conduction band (*E*_CB_) potential.^59^ Therefore, the *E*_CB_ value of TiO_2_/Ti_3_CNT_*x*_ can be determined to be −0.80 V *vs* NHE. The more negative value of *E*_CB_ compared to the redox potential of O_2_/O_2_^–•^ (−0.32 *vs* NHE) suggests that TiO_2_/Ti_3_CNT_*x*_ can reduce adsorbed O_2_ to O_2_^–•^.^60^ Furthermore, by taking the band gap of anatase TiO_2_ (3.2 eV),^61^ the valence band (*E*_VB_) potential is estimated to be at 2.4 V *vs* NHE. This is slightly lower than the redox potential of ^•^OH/OH^–^ (2.38 V *vs* NHE), indicating the capability of holes in TiO_2_/Ti_3_CNT_*x*_ to produce reactive ^•^OH radicals.^62,63^

**Figure 6:**
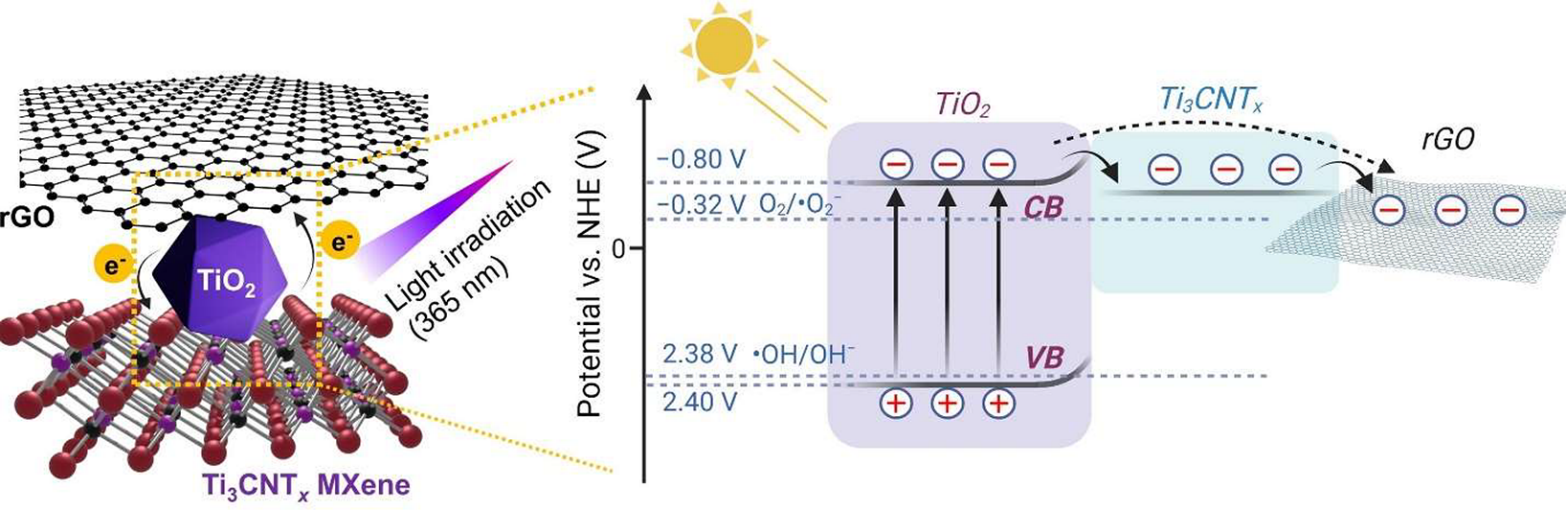
Schematic image illustrating photogenerated electron transfer at rGO/Ti_3_CNT_*x*_ interface under UV light irradiation.

The optoelectronic characterization and the Mott–Schottky analysis further allowed us to propose the photogenerated charge transfer mechanism in the rGO/Ti_3_CNT_*x*_ thin film. Figure 6 shows the charge transfer process in the rGO/Ti_3_CNT_*x*_ thin film upon UV irradiation. During UV irradiation, interfacial TiO_2_ on the surface of Ti_3_CNT_*x*_ produces photogenerated electron–hole pairs in the conduction and valence bands of TiO_2_, respectively. The electrons are further transferred to electrically conductive Ti_3_CNT_*x*_ owing to an intimate interface and band alignment at the metal–semiconductor interface. As TiO_2_ is a wide-band-gap n-type semiconductor, an electron-blocking Schottky barrier is expected to form, resulting in a more efficient separation of photogenerated charge carriers.^64^ rGO acts as an additional conductive channel to capture the photogenerated electrons from the TiO_2_/Ti_3_CNT_*x*_ interface. Based on the Mott–Schottky analysis, the photogenerated electrons at *E*_CB_ and holes at *E*_VB_ of interfacial TiO_2_ can produce reactive oxygen species (^•^O_2_^–^ and OH), which further react with and decompose the dye.

According to the optoelectrical studies above, the rGO/Ti_3_CNT_*x*_ thin film is suitable as a transparent coating with sufficient conductivity and self-cleaning properties. Most importantly, adding rGO layers on top of a Ti_3_CNT_*x*_ thin film makes the coating more resistant to degradation, thereby increasing its environmental stability when exposed to the ambient atmosphere while having no measurable influence on the optoelectronic properties of the MXene film. The superior optoelectronic properties and increased environmental stability open avenues for the applications of rGO/Ti_3_CNT_*x*_ thin films as coatings.

## 4 Conclusions

In conclusion, we fabricated multifunctional rGO/Ti_3_CNT_*x*_ heterostructured thin films under ambient conditions *via* a layer-by-layer (LbL) spin-coating method. The optical transparency and conductivity of thin films can be easily controlled by adjusting the number of spin-coating cycles. We demonstrated that rGO/Ti_3_CNT_*x*_ thin films exhibit an efficient dye discoloration ability and high optical transparency, as well as good electrical conductivity, making them promising for various applications, such as self-cleaning front contacts in photovoltaic solar cells. The combination of these properties is unusual but essential to minimize the buildup of contaminants and ensure the film can transport electricity efficiently.

We revealed that adding the rGO barrier layer enhances the environmental stability of Ti_3_CNT_*x*_ layers when exposed to ambient air for 21 days. We also investigated the photosensitivity of the rGO/Ti_3_CNT_*x*_ thin films and demonstrated that rGO can aid Ti_3_CNT_*x*_ in extracting photogenerated charge carriers more efficiently, thus facilitating more effective dye photocatalytic discoloration under UV irradiation. The fabricated rGO/Ti_3_CNT_*x*_ thin films show potential as multifunctional transparent conductive coatings in various applications, including flat panel displays and photovoltaic devices.

## Supporting Information Available

Additional details of experimental procedures, SEM images and UV–vis, FTIR, and Raman spectra of GO flakes, XRD spectra of Ti_3_AlCN MAX phase and SL Ti_3_CNT_*x*_, EDS spectra and mapping of SL Ti_3_CNT_*x*_, zeta potential, UV–vis absorption spectra, and calculated mass extinction coefficient of SL Ti_3_CNT_*x*_ aqueous dispersion, calculation of SL Ti_3_CNT_*x*_ thin films figure of merit, oxidation stability test of Ti_3_CNT_*x*_ and GO/Ti_3_CNT_*x*_ thin films, SEM and STEM images of rGO/Ti_3_CNT_*x*_ thin films, *I*–*V* curve of Ti_3_CNT_*x*_ and rGO/Ti_3_CNT_*x*_ thin films after 7 months of storage, sheet resistance value of GO/Ti_3_CNT_*x*_ thin films, photocurrent density of Ti_3_CNT_*x*_, rGO, rGO/Ti_3_CNT_*x*_, and GO/Ti_3_CNT_*x*_ under different wavelengths, digital photograph of RhB photolytic discoloration, RhB discoloration kinetics under UV light and white light irradiation, photostability of rGO/Ti_3_CNT_*x*_ under UV irradiation, Mott–Schottky plot of rGO/Ti_3_CNT_*x*_, atomic and weight percentage of chemical elements identified by EDS for SL-Ti_3_CNT_*x*_, and comparison of electrical and optical properties of photocatalytic thin films reported in the literature (PDF)
