# Supplementary material for: Ambient Processed rGO/Ti3CNTx MXene Thin Film with High Oxidation Stability, Photosensitivity, and Self-Cleaning Potential
Source: ACS Appl Mater Interfaces. 2023 Sep 8;15(37):44075–86. doi: 10.1021/acsami.3c07972 (PMC10520912; doi:10.1021/acsami.3c07972)
Supplement: Supplementary file 1 — am3c07972_si_001.pdf [file am3c07972_si_001.pdf]

Supporting Information

Ambient Processed rGO/Ti_3CNT_x MXene Thin Film with High Oxidation Stability, Photosensitivity, and Self-Cleaning Potential

Muhammad Abiyyu Kenichi Purbayanto[1,*] Dominika Bury[1], Madhurya Chandel[1], Zhila Dehghan Shahrak[2], Vadym N. Mochalin[2,3], Anna Wójcik[4], Dorota Moszczyńska[1], Anita Wojciechowska[1], Anika Tabassum[5], Michael Naguib[5], Agnieszka Maria Jastrzębska[1*]

[1] Faculty of Materials Science and Engineering, Warsaw University of Technology, Wołoska 141, 02-507, Poland

[2] Department of Chemistry, Missouri University of Science and Technology, Rolla, Missouri 65409 United States

[3] Department of Materials Science and Engineering, Missouri University of Science and Technology, Rolla, Missouri 65409 United States

[4] Polish Academy of Sciences, Institute of Metallurgy and Materials Science, W. Reymonta 25, 30-059 Cracow, Poland

[5] Department of Physics and Engineering Physics, Tulane University, New Orleans, Louisiana 70118, United States

*email: muhammad_abiyyu.kenichi.dokt@pw.edu.pl, agnieszka.jastrzebska@pw.edu.pl

Experimental procedures

Preparation of reduced graphene oxide (rGO) referential samples

To study the optoelectronic properties of rGO alone, we fabricated rGO thin film by using a mixture of graphene oxide (4 mg/mL) and L-ascorbic acid (50 mM) with a volume ratio of 1:1. The mixture was further drop-casted on the activated glass substrate with the volume of 30 μL and heated at 150°C for 20 minutes in ambient condition. Here, we used the drop-casting technique, as layer-by-layer spin coating is unsuitable for depositing rGO due to its hydrophobic nature.

Studied on the electrochemical properties

To study the flat band potential, we performed Mott-Schottky (MS) measurements using an electrochemical workstation (VSP-300, Biologic, France). The prepared samples were coated on glassy carbon as a working electrode, while Ag/AgCl (saturated in KCl) and a platinum wire were used as reference and counter electrodes, respectively. Here, 0.1 M KCl was used as a supporting electrolyte. To prepare the working electrode, the samples were sonicated for 30 minutes. Then 10 μL of the solution is drop-casted on the GC and dried overnight at the ambient temperature. MS measurement analysis was done under the frequency of 1 kHz. All the measurements were done in the dark at room temperature.

Supplementary Figures

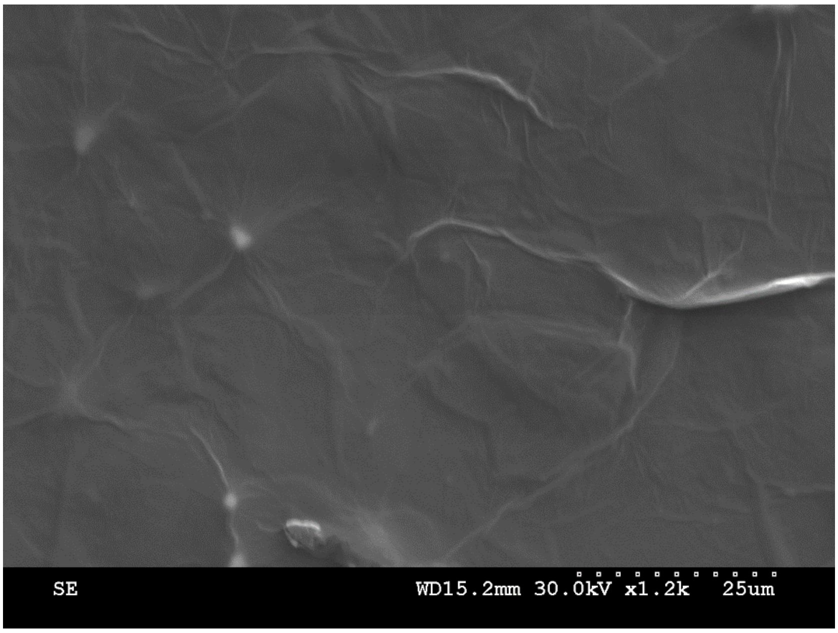

Figure S1. SEM image of GO flakes obtained by a modified Hummer's method.

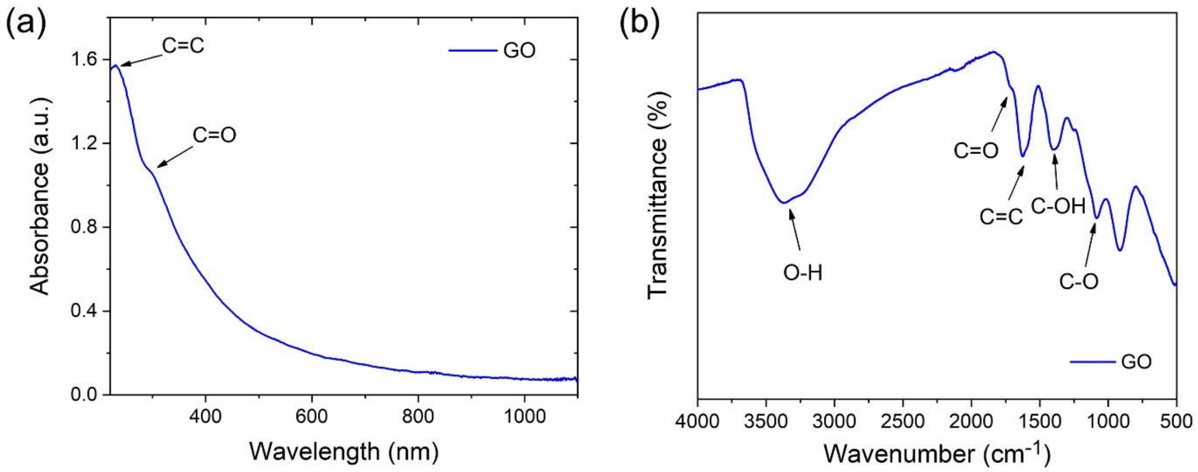

Figure S2. (a) UV-Vis and (b) ATR-FTIR spectra of GO flakes.

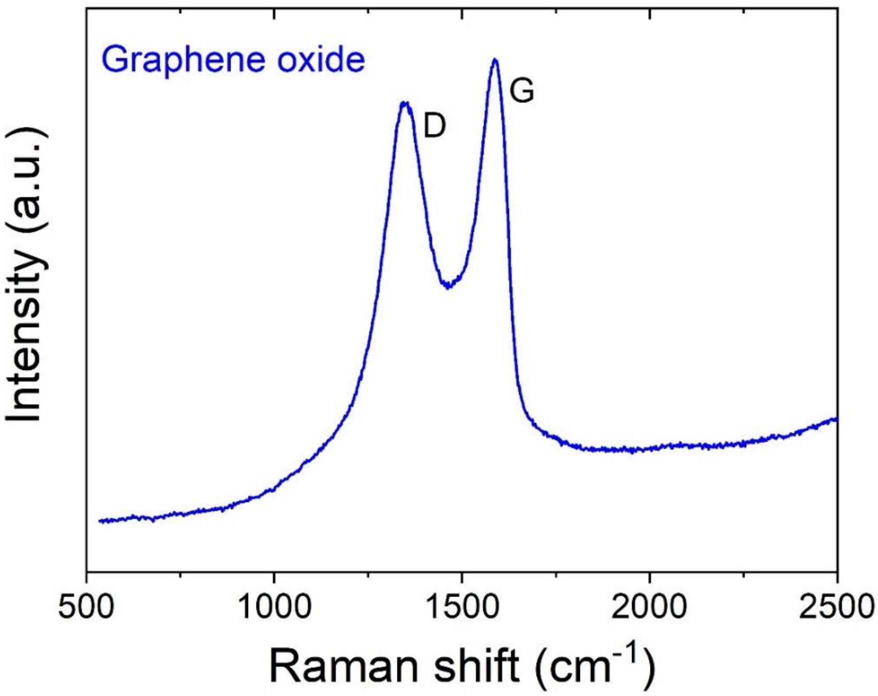

Figure S3. Raman spectra of GO flakes.

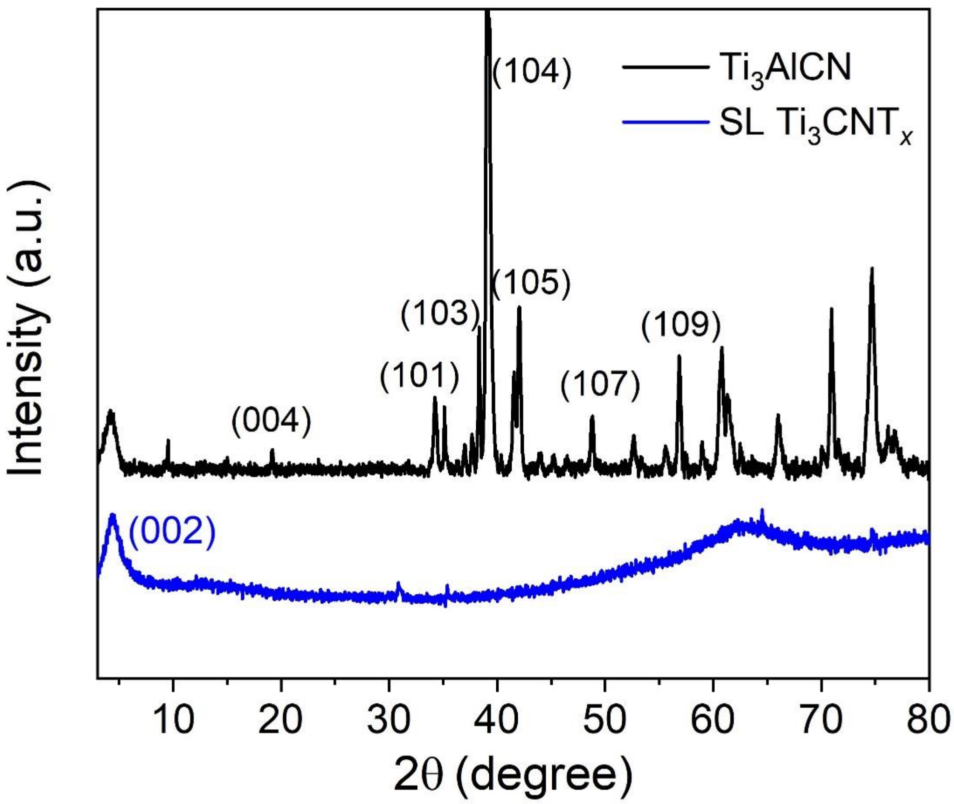

Figure S4. XRD spectra of Ti_3AlCN MAX phase and SL Ti_3CNT_x obtained *via* microwave-assisted hydrothermal reaction.

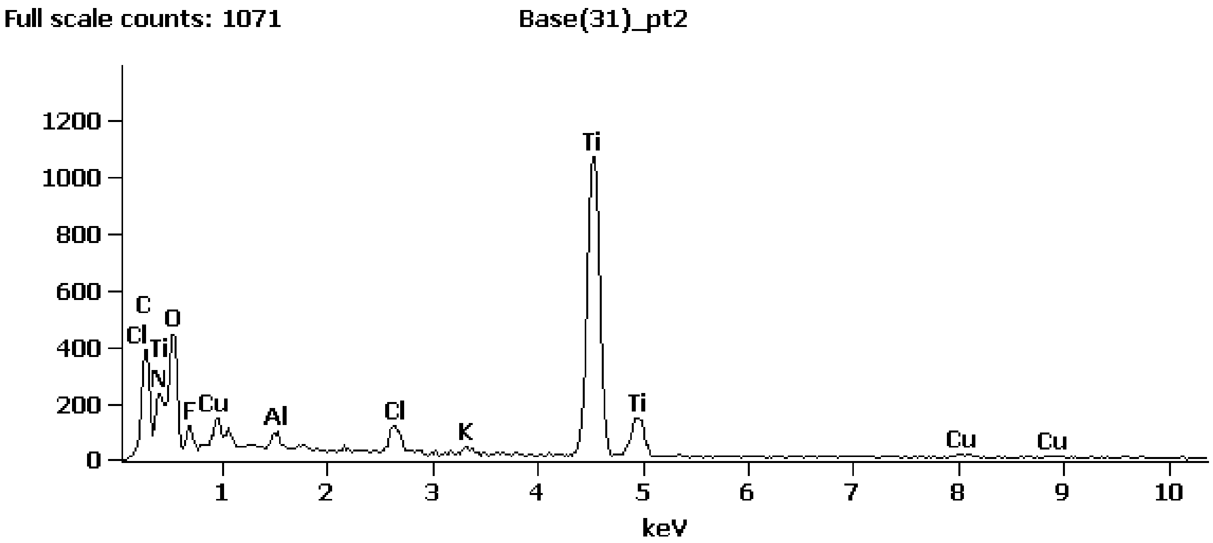

Figure S5. EDS spectra of SL Ti_3CNT_x MXene

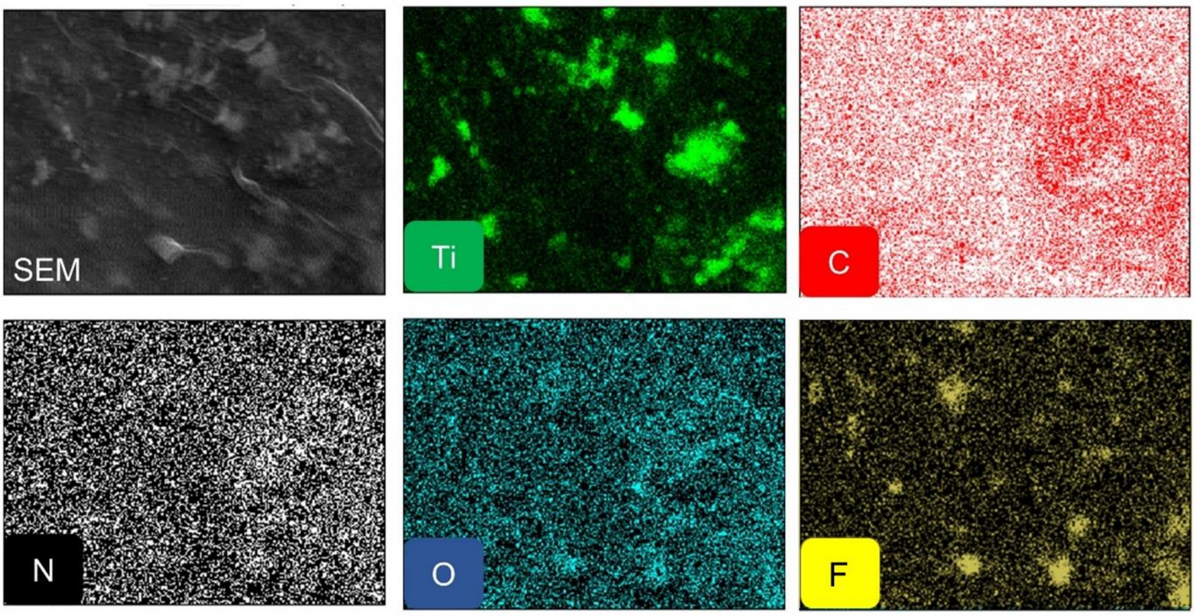

Figure S6. EDS mapping of SL Ti_3CNT_x dispersion drop-casted on Si substrate.

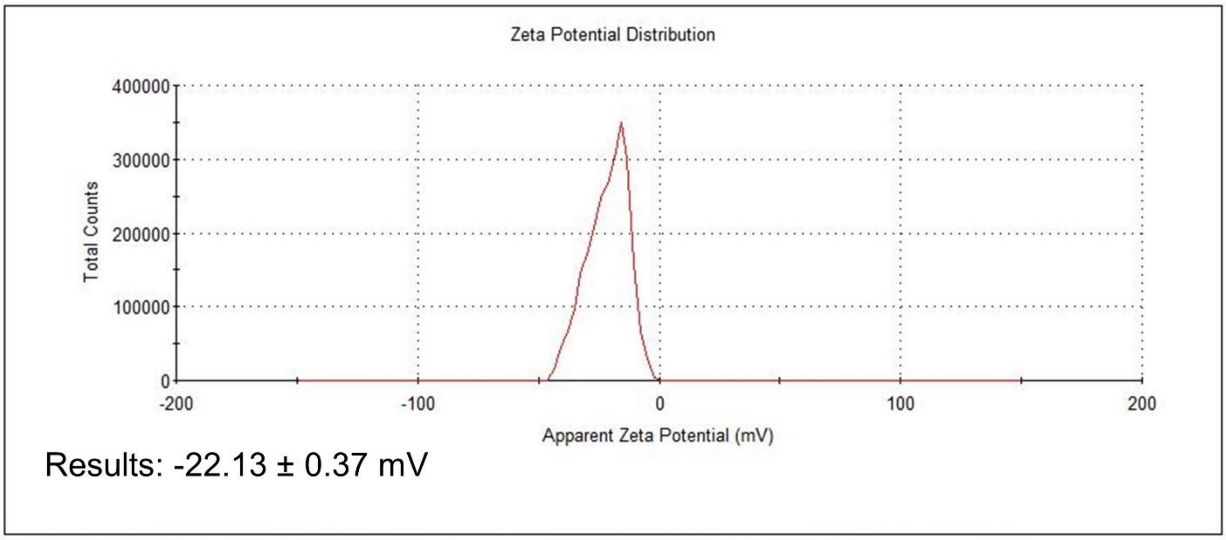

Figure S7. The Zeta potential distribution graph of SL Ti_3CNT_x

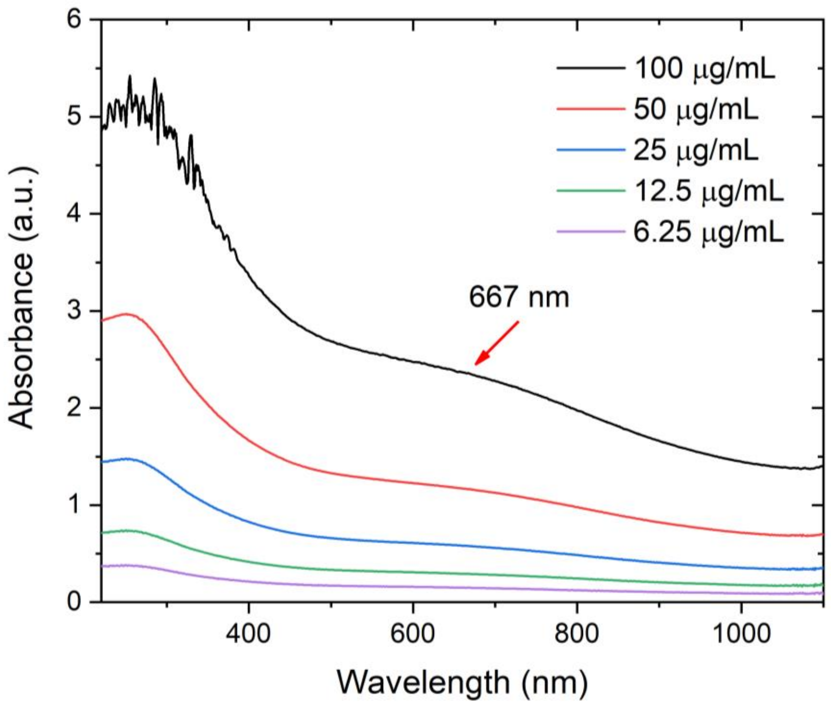

Figure S8. UV-Visible absorption spectra of SL Ti_3CNT_x aqueous dispersion with various concentrations.

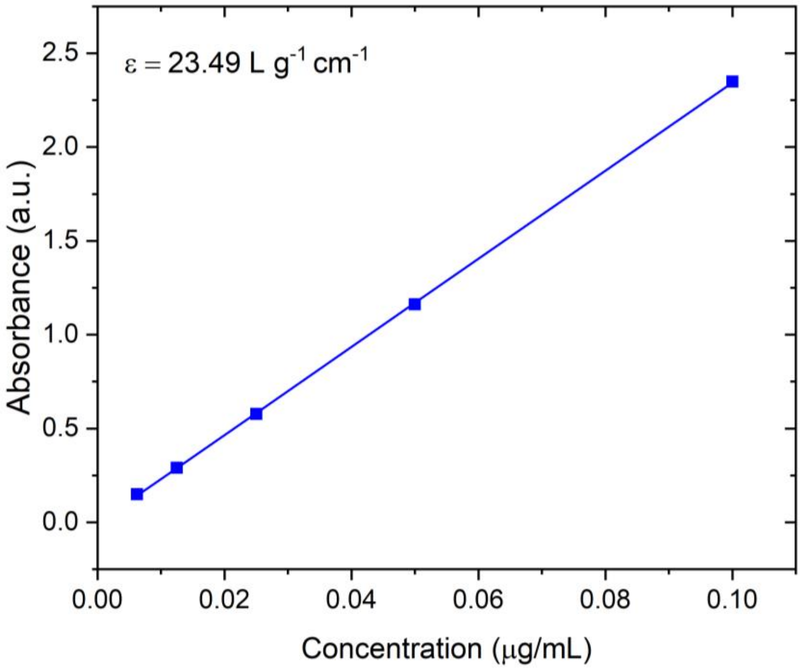

Figure S9. The calculation of the mass extinction coefficient of SL Ti_3CNT_x. Here, the calibration curves of the solution were obtained by taking the peak absorption maxima of Ti_3CNT_x at the visible region (667 nm).

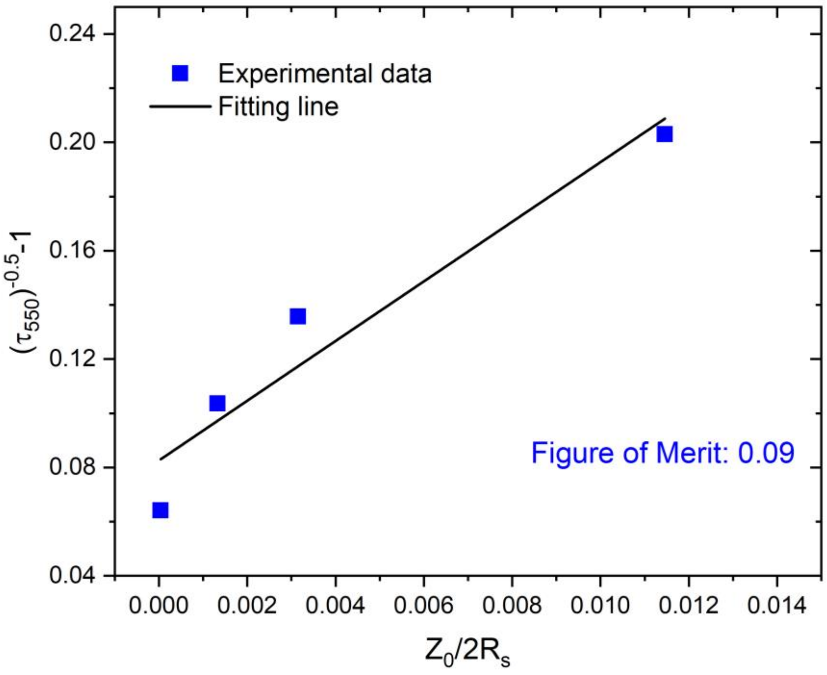

Figure S10. The calculation of Ti_3CNT_x thin films figure of merit obtained by taking the slope of $(\tau_{550})^{-0.5}$-1 and vs $Z_0/2R_s$ plot.

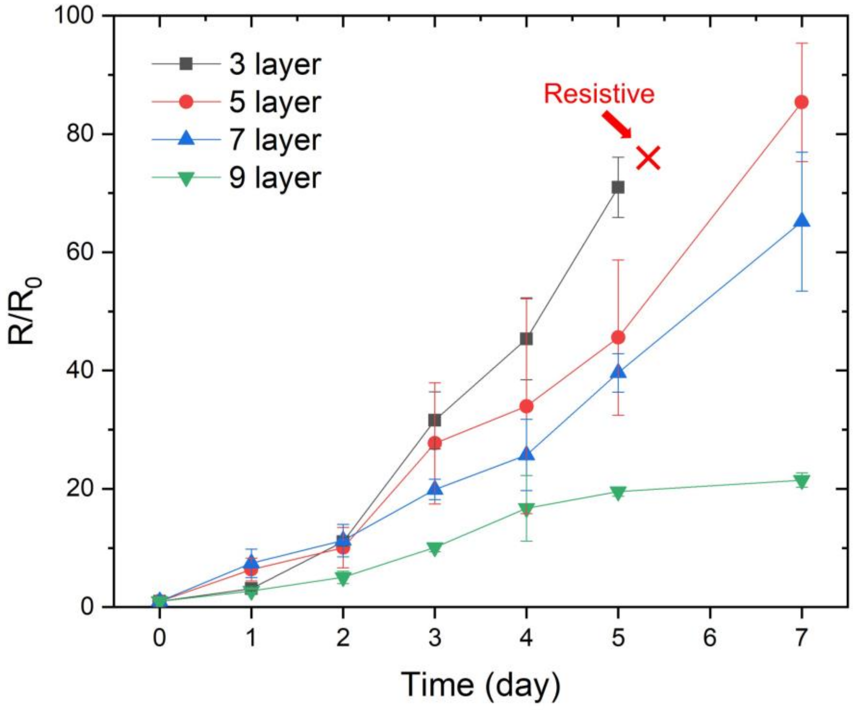

Figure S11. The oxidation stability test of Ti_3CNT_x thin films spin-coated with an increasing number of cycles. The test was conducted by exposing the films for seven days.

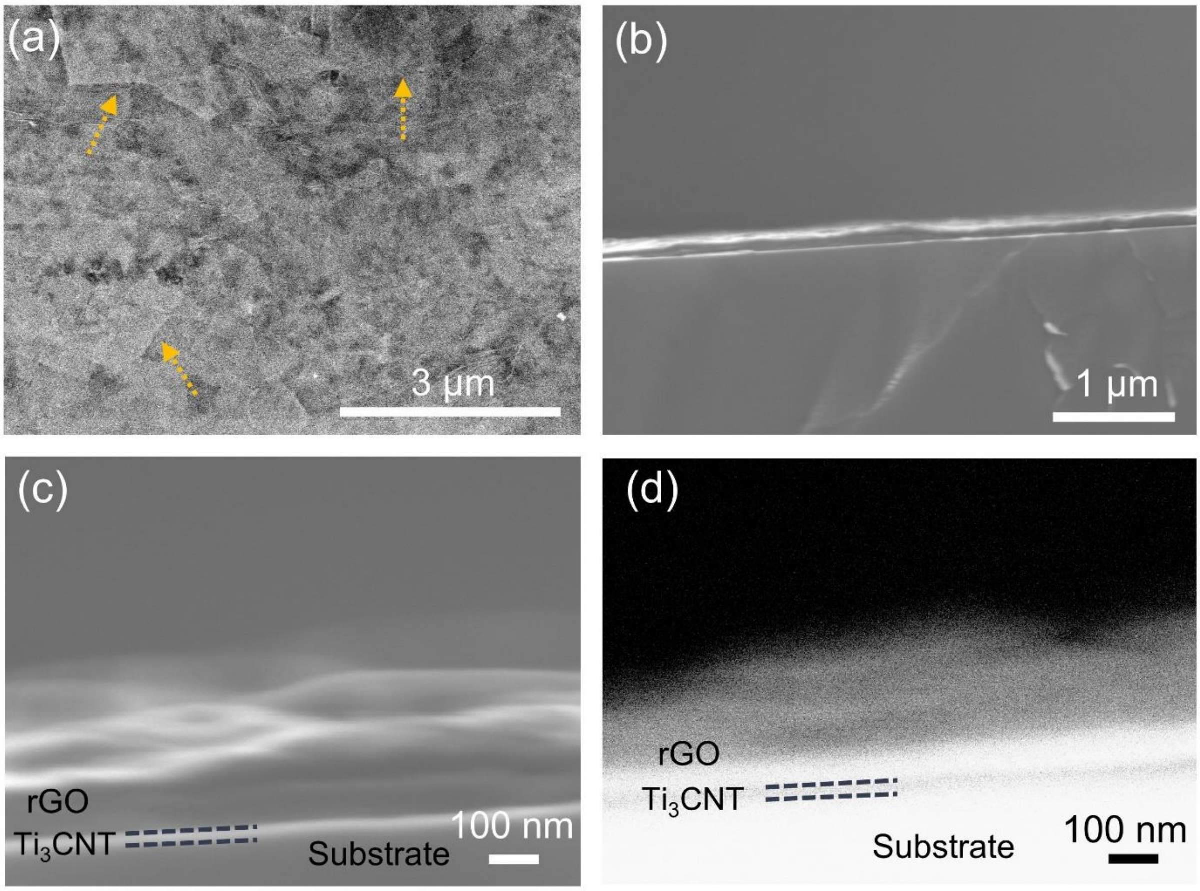

Figure S12. (a) Surface morphology image, (b) cross-sectional image, and (c) the corresponding magnified image of rGO/Ti_3CNT_x thin films on Si substrate. (d) backscattered electron image of rGO/Ti_3CNT_x thin film. Yellow arrows indicated Ti_3CNT_x flakes covered by rGO flakes.

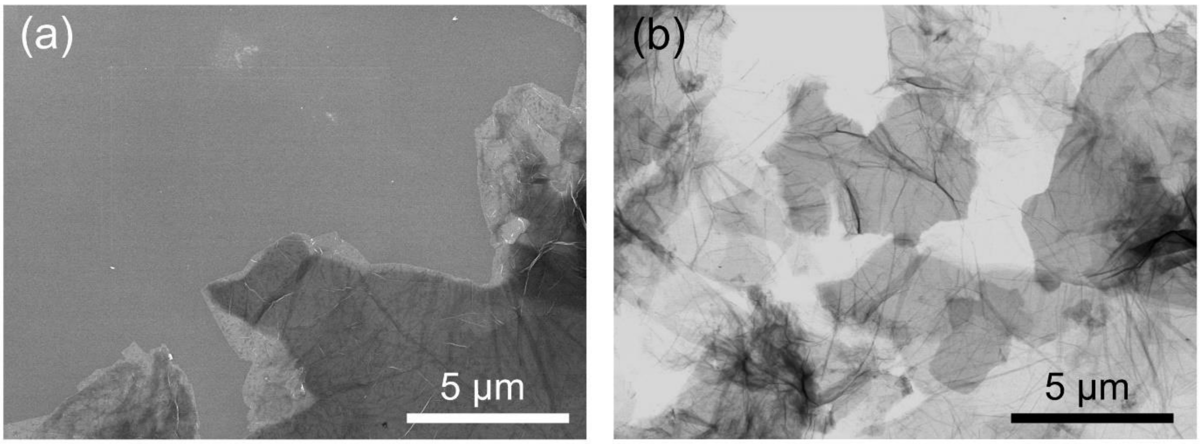

Figure S13. (a) SEM and (b) scanning tunneling electron microscopy images showing the interfacial connection between rGO and Ti_3CNT_x.

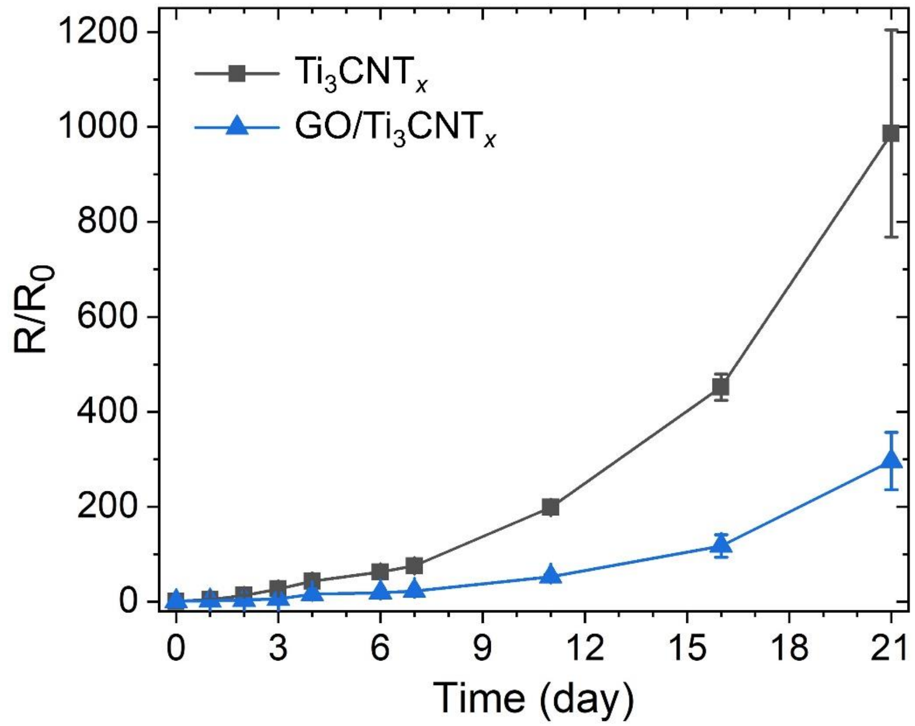

Figure S14. Sheet resistance changes of Ti_3CNT_x and GO/Ti_3CNT_x thin films measured up to 21 days of storage.

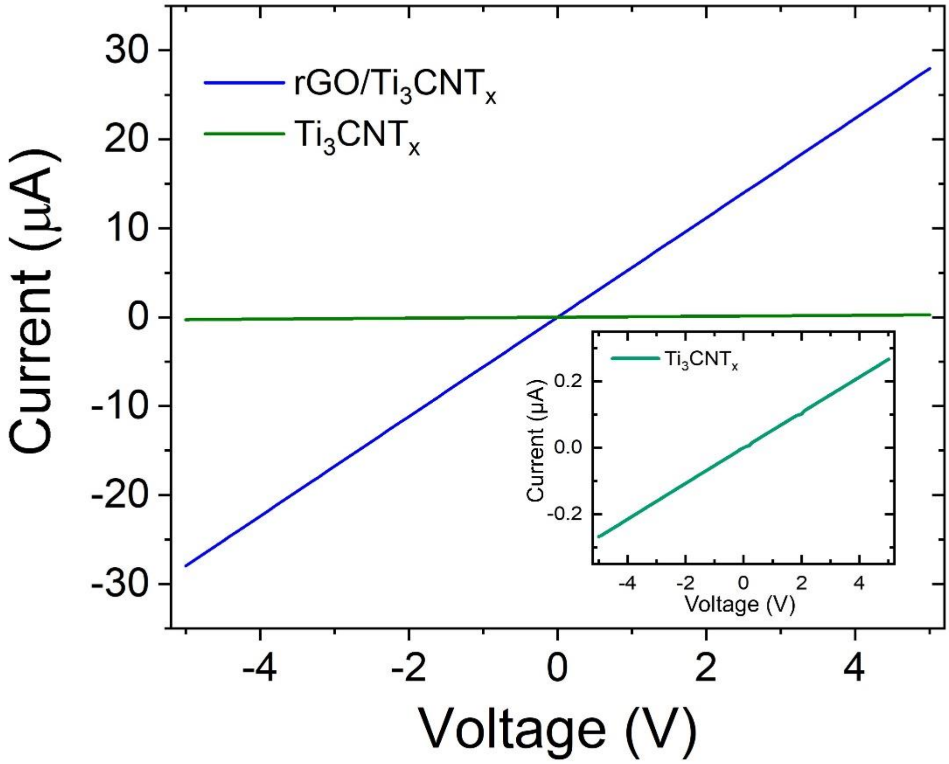

Figure S15. I-V curve of rGO/Ti_3CNT_x and Ti_3CNT_x thin films after 7 months of storage. The inset shows the magnified curve of Ti_3CNT_x.

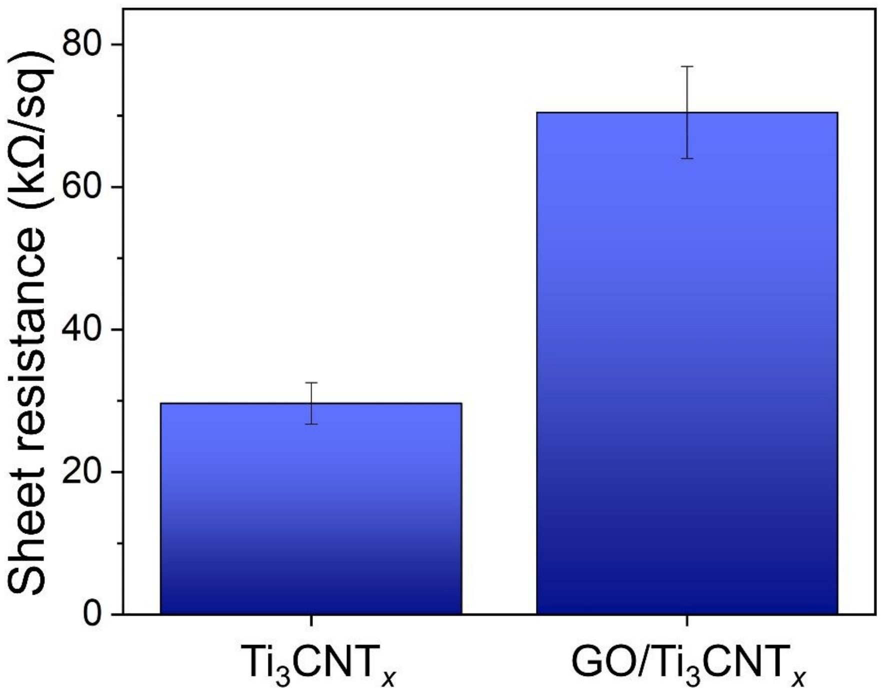

Figure S16. Sheet resistance value of Ti_3CNT_x and GO/Ti_3CNT_x thin films.

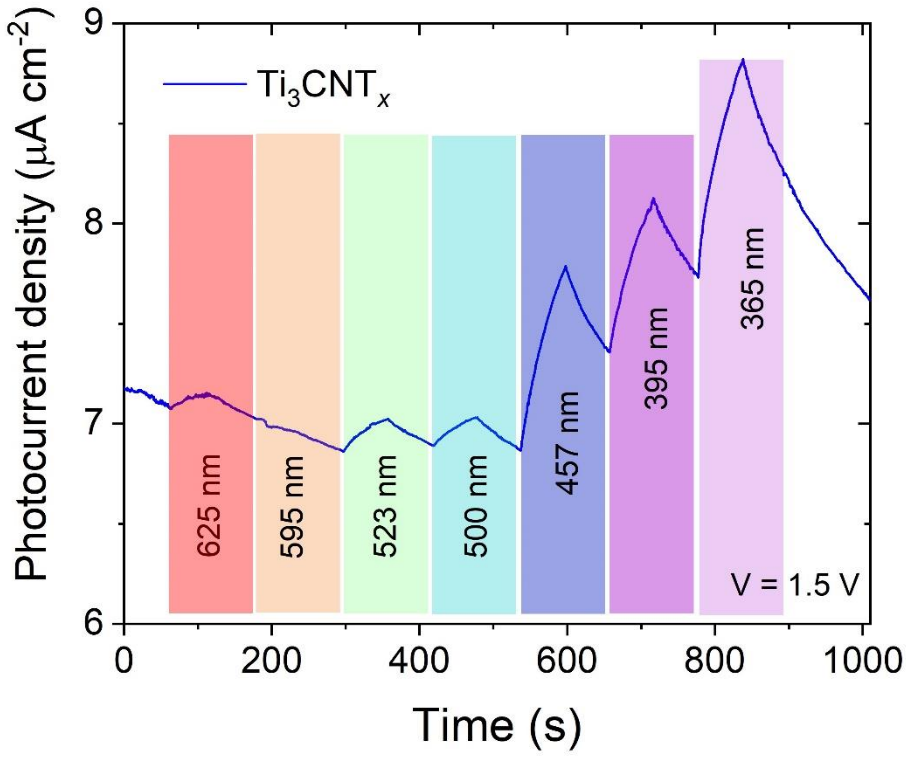

Figure S17. Photocurrent density of Ti_3CNT_x thin film upon irradiation by different wavelengths. The bias voltage of 1.5V was used.

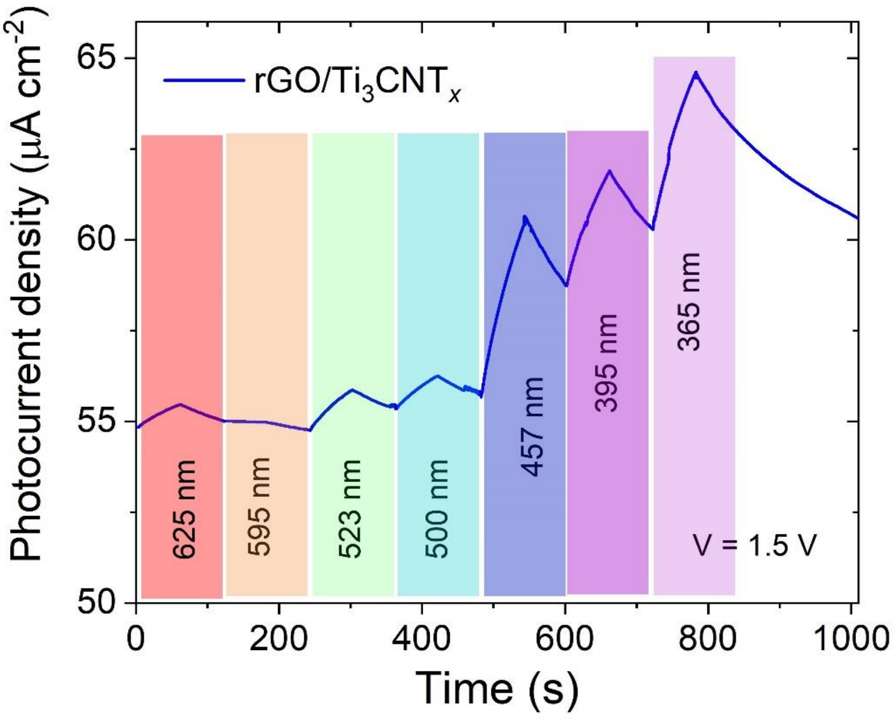

Figure S18. Photocurrent density of rGO/Ti_3CNT_x thin film upon irradiation by different wavelengths. The bias voltage of 1.5V was used.

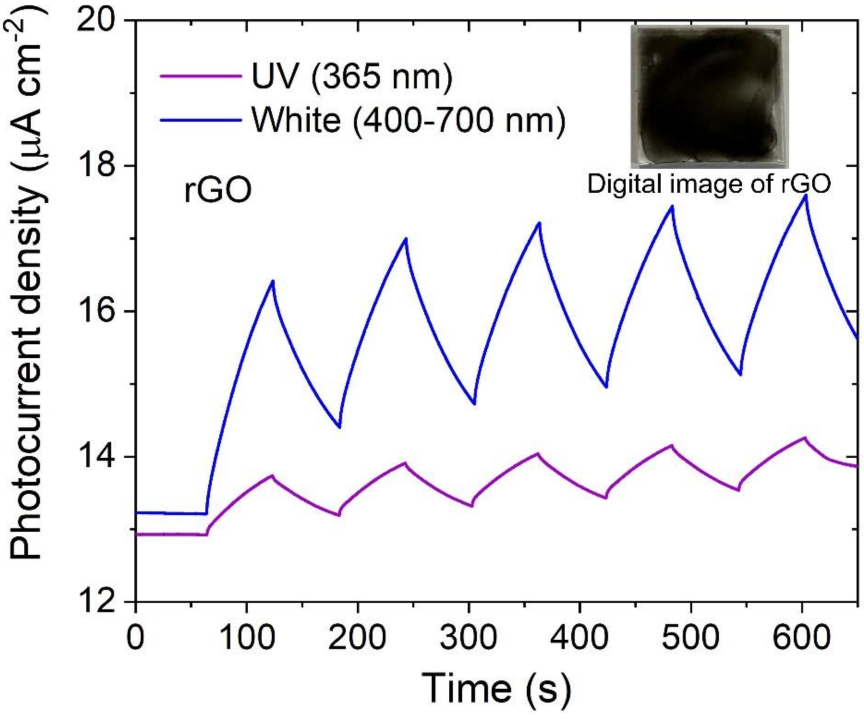

Figure S19. Photocurrent density of rGO thin film upon UV and white light irradiation. The inset shows the digital photograph of rGO thin film deposited on the glass substrate for the photocurrent test. The bias voltage of 1.5V was used.

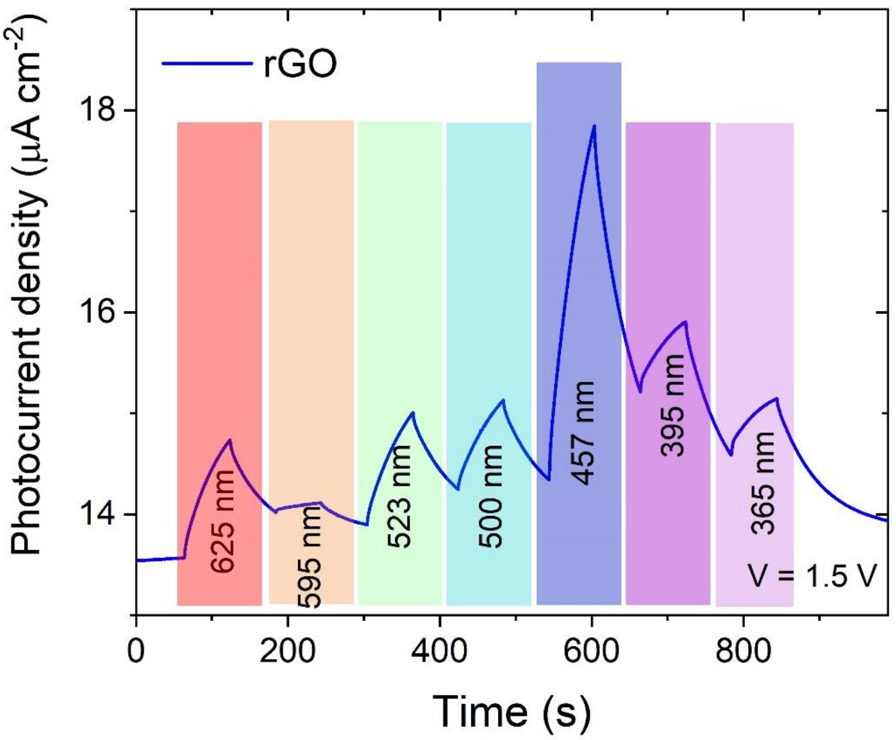

Figure S20. Photocurrent density of rGO thin film upon irradiation by different wavelengths. The bias voltage of 1.5V was used.

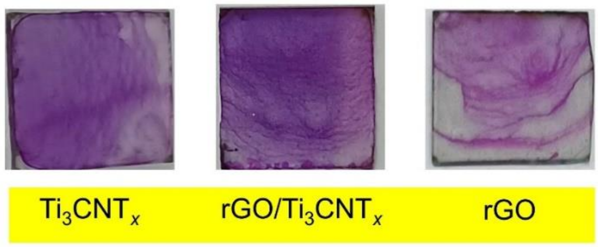

Figure S21. RhB adsorption on the surface of Ti_3CNT_x, rGO/Ti_3CNT_x, and bare rGO thin film.

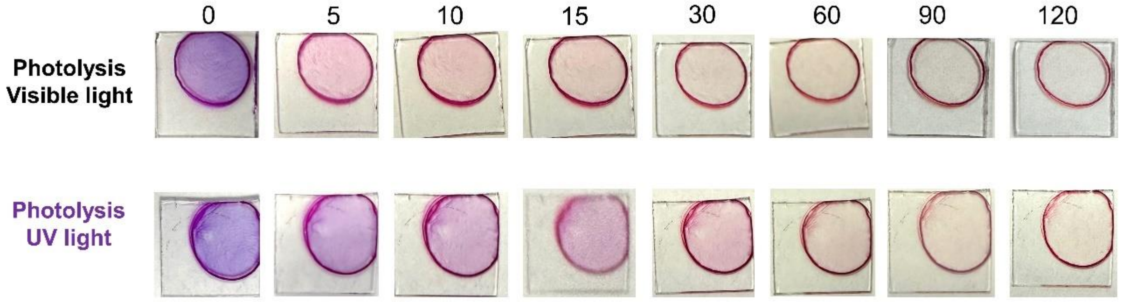

Figure S22. Digital photograph of RhB photolytic discoloration under visible and UV light.

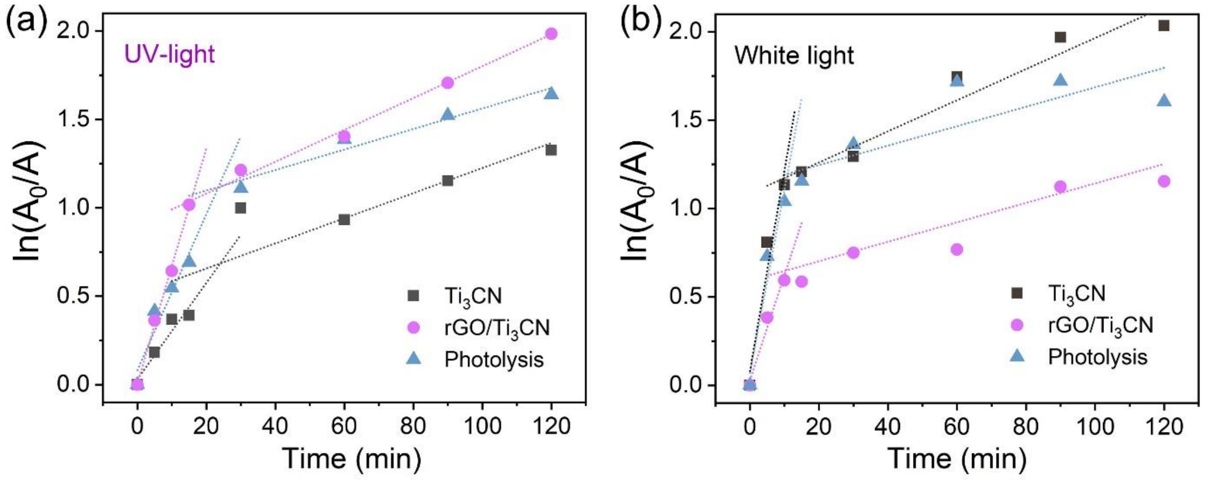

Figure S23. RhB discoloration kinetics under (a) UV light and (b) white light irradiation performed up to 120 minutes. Here, the pseudo-first-order kinetic model is used.[1]

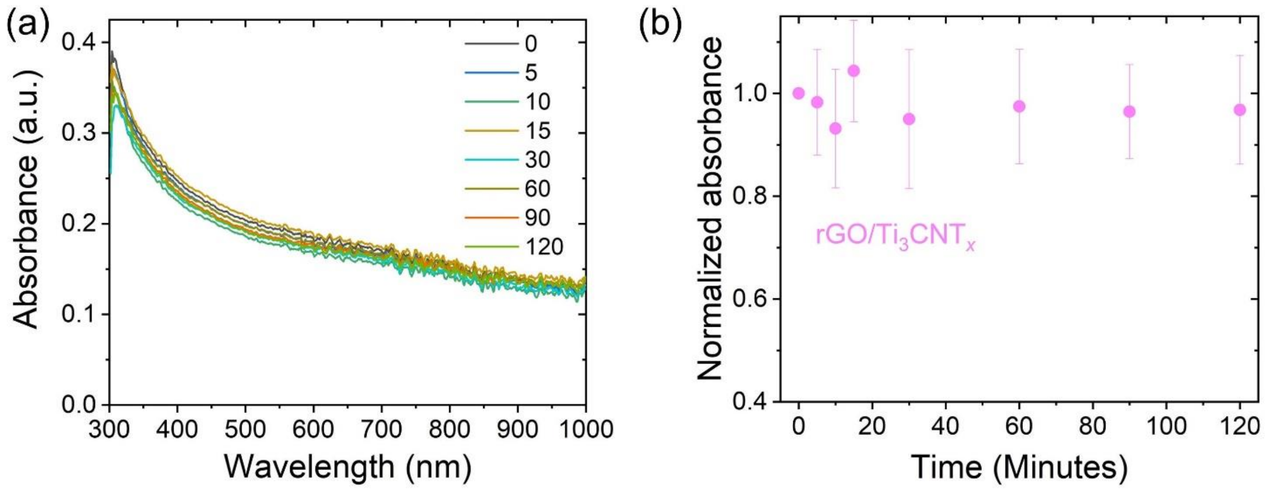

Figure S24. (a) UV-Vis absorption spectra and (b) change in the absorbance at 667 nm of rGO/Ti_3CNT_x under different UV irradiation times.

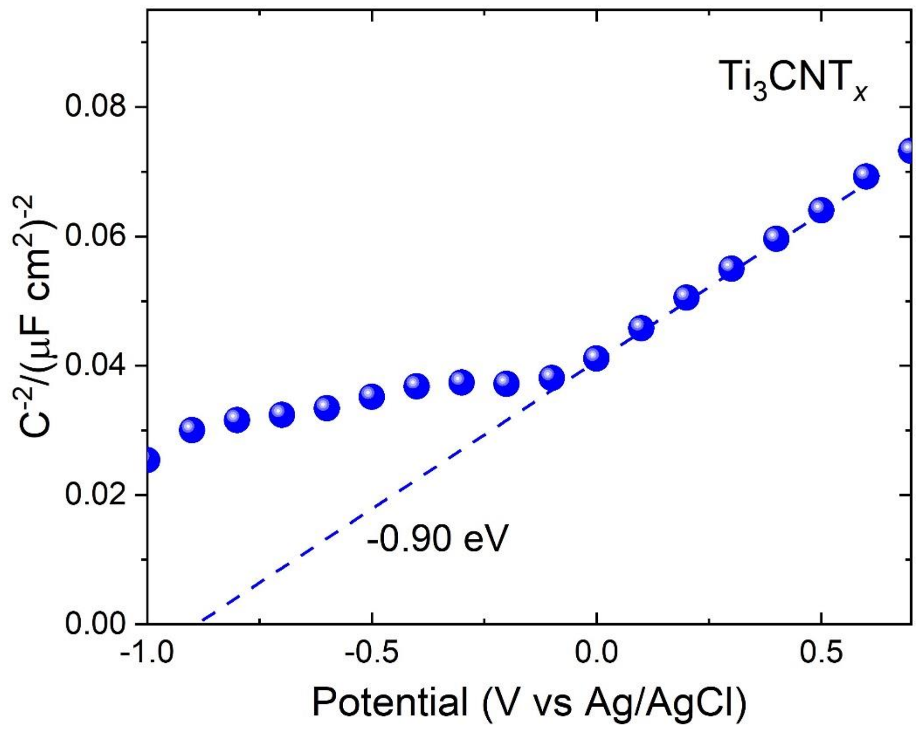

Figure S25. The Mott-Schottky plot of Ti_3CNT_x at the frequency of 1 kHz.

Supplementary Tables

Table S1. Atomic and weight percentage of chemical elements identified by EDS for SL Ti_3CNT_x samples.

Element	At.%	Wt.%
Titanium	15.41 ± 0.17	38.60 ± 0.43
Carbon*	59.28 ± 1.46	37.23 ± 0.92
Nitrogen	8.99 ± 1.07	6.58 ± 0.79
Oxygen	11.21 ± 0.33	9.38 ± 0.27
Fluorine	2.34 ± 0.19	2.33 ± 0.19
Chlorine	1.23 ± 0.05	2.27 ± 0.09
Aluminum	0.60 ± 0.09	0.85 ± 0.12
Copper*	0.67 ± 0.12	2.22 ± 0.40
Potassium	0.26 ± 0.03	0.54 ± 0.07

*: Observable Cu and excessive C contributions come from carbon coated copper TEM grid.

Potassium contribution in EDS comes from the impurity.

Table S2. The comparison of electrical and optical properties of photocatalytic thin films reported in the literature.

Material	Fabrication method	Transmittance at 550 nm (%)	Sheet resistance (Ω/sq)	Reference
Ti_3CNT_x	Spin coating	88	4,560,000	This work
Ti_3CNT_x		82	141,505	This work
Ti_3CNT_x		78	59,664	This work
Ti_3CNT_x		69	16,444	This work
rGO/Ti_3CNT_x		74	19,393	This work
Nb-doped TiO_2	Spin coating	47	1,200,000	[2]
Graphene/TiO_2	Spray coating	Between 80 to 10	~20,000,000	[3]
Multiwalled carbon nanotubes (MWCNT)/TiO_2	Layer-by-Layer assembly	Not determined	In the range of 10^8 to 10^5	[4]
Multiwalled carbon nanotubes (MWCNT)/TiO_2	Spin coating	37	33,000	[5]
p-type NiO	Radio frequency sputtering	46	7,950,000	[6]
Black TiO_2	Magnetron sputtering + hydrogen plasma	~55	1,930	[7]
ZnO	Chemical vapor deposition	68	15,053	[8]
Cl-doped ZnO		82	1,064	[8]

References

(1) Bury, D.; Jakubczak, M.; Purbayanto, M. A. K.; Wojciechowska, A.; Moszczyńska, D.; Jastrzębska, A. M. Photocatalytic Activity of the Oxidation Stabilized $Ti_3C_2T_x$ MXene in Decomposing Methylene Blue, Bromocresol Green and Commercial Textile Dye. *Small Methods* **2023**, 2201252.

(2) Fallah, M.; Zamani-Meymian, M.-R.; Rahimi, R.; Rabbani, M. Effect of Annealing Treatment on Electrical and Optical Properties of Nb Doped TiO_2 Thin Films as a TCO Prepared by Sol–Gel Spin Coating Method. *Appl. Surf. Sci.* **2014**, *316*, 456–462.

(3) Zabihi, F.; Ahmadian-Yazdi, M.-R.; Eslamian, M. Photocatalytic Graphene-TiO_2 Thin Films Fabricated by Low-Temperature Ultrasonic Vibration-Assisted Spin and Spray Coating in a Sol-Gel Process. *Catalysts* **2017**, *7* (5), 136.

(4) Tettey, K. E.; Yee, M. Q.; Lee, D. Photocatalytic and Conductive MWCNT/TiO_2 Nanocomposite Thin Films. *ACS Appl. Mater. Interfaces* **2010**, *2* (9), 2646–2652.

(5) Castro, M.; Oliveira, P.; Schmidt, H. Optical, Structural and Electrical Investigations of TiO_2/Multi-Walled Carbon Nanotube Composites. *J. Nanosci. Nanotechnol.* **2009**, *9* (7), 4016–4021.

(6) Oh, J.-H.; Hwang, S. Y.; Kim, Y. D.; Song, J.-H.; Seong, T.-Y. Effect of Different Sputtering Gas Mixtures on the Structural, Electrical, and Optical Properties of p-Type NiO Thin Films. *Mater. Sci. Semicond. Process.* **2013**, *16* (5), 1346–1351.

(7) Godoy Junior, A.; Pereira, A.; Gomes, M.; Fraga, M.; Pessoa, R.; Leite, D.; Petraconi, G.; Nogueira, A.; Wender, H.; Miyakawa, W. Black TiO_2 Thin Films Production Using Hollow Cathode Hydrogen Plasma Treatment: Synthesis, Material Characteristics and Photocatalytic Activity. *Catalysts* **2020**, *10* (3), 282.

(8) Jiamprasertboon, A.; Powell, M. J.; Dixon, S. C.; Quesada-Cabrera, R.; Alotaibi, A. M.; Lu, Y.; Zhuang, A.; Sathasivam, S.; Siritanon, T.; Parkin, I. P. Photocatalytic and Electrically Conductive Transparent Cl-Doped ZnO Thin Films via Aerosol-Assisted Chemical Vapour Deposition. *J. Mater. Chem. A* **2018**, *6* (26), 12682–12692.
